# Selection on Expected Maximum Haploid Breeding Values Can Increase Genetic Gain in Recurrent Genomic Selection

**DOI:** 10.1534/g3.118.200091

**Published:** 2018-02-06

**Authors:** Dominik Müller, Pascal Schopp, Albrecht E. Melchinger

**Affiliations:** * University of Hohenheim, Institute of Plant Breeding, Seed Science and Population Genetics, Fruhwirthstr. 21, 70599 Stuttgart and; † KWS SAAT SE, Grimsehlstraβe 31, 37574 Einbeck

**Keywords:** genetic gain, doubled haploid, optimal haploid value, expected maximum haploid breeding value, GenPred, Shared Data Resources, Genomic Selection

## Abstract

Genomic selection (GS) offers the possibility to estimate the effects of genome-wide molecular markers, which can be used to calculate genomic estimated breeding values (GEBVs) for individuals without phenotypes. GEBVs can serve as a selection criterion in recurrent GS, maximizing single-cycle but not necessarily long-term genetic gain. As simple genome-wide sums, GEBVs do not take into account other genomic information, such as the map positions of loci and linkage phases of alleles. Therefore, we herein propose a novel selection criterion called expected maximum haploid breeding value (EMBV). EMBV predicts the expected performance of the best among a limited number of gametes that a candidate contributes to the next generation, if selected. We used simulations to examine the performance of EMBV in comparison with GEBV as well as the recently proposed criterion optimal haploid value (OHV) and weighted GS. We considered different population sizes, numbers of selected candidates, chromosome numbers and levels of dominant gene action. Criterion EMBV outperformed GEBV after about 5 selection cycles, achieved higher long-term genetic gain and maintained higher diversity in the population. The other selection criteria showed the potential to surpass both GEBV and EMBV in advanced cycles of the breeding program, but yielded substantially lower genetic gain in early to intermediate cycles, which makes them unattractive for practical breeding. Moreover, they were largely inferior in scenarios with dominant gene action. Overall, EMBV shows high potential to be a promising alternative selection criterion to GEBV for recurrent genomic selection.

The identification, selection and propagation of superior individuals builds the foundation of all breeding efforts. The breeding potential of a candidate is classically determined by its breeding value (BV), the sum of all additive effects at quantitative trait loci (QTL) affecting a complex trait (Lynch and Walsh 1998). While BVs have been estimated in progeny tests, Meuwissen *et al.* (2001) proposed genomic selection (GS) to predict BVs prior to phenotypic evaluation. The principle is to use genome-wide marker data and phenotypes of training individuals to calculate locus-specific allele substitution effects. Genomic estimated breeding values (GEBVs) are then calculated as predictors of BVs. Selecting individuals ranked according to their BVs maximizes the population mean of the next cycle when they are recombined, but a repeated application of this selection strategy does not necessarily maximize long-term genetic gain over several generations (Wray and Goddard 1994; Liu *et al.* 2015). GEBVs, as predictors of BVs, are subject to the same constraints. This suboptimal behavior can be explained by the fact that GEBVs are simple genome-wide sums of estimates of allele substitution effects, which can conceal the contribution of favorable alleles with small effects. The later are less relevant for short-term gain and can be easily lost, especially if their frequency is low, but can play an important role for long-term gain by maintaining useful genetic variance (Jannink 2010; Liu *et al.* 2015).

To prevent the loss of rare favorable alleles, Goddard (2009) proposed a modified GEBV that weights estimated allele substitution effects using the frequencies of favorable alleles, such that rare alleles receive a higher weight. This criterion does not take into account the magnitude of the effects based on the premise that, for optimal long-term genetic gain, all favorable alleles should ultimately be fixed. Later, Jannink (2010) suggested a modification called weighted GS, herein referred to as weighted GEBV (wGEBV). This considers also the magnitude of the effects, because especially for QTL with small effects determining which allele is the favorable one is problematic. wGEBV proved to be superior to GEBVs in terms of long-term genetic gain (19 cycles) in spring barley (*Hordeum vulgare* L., Jannink 2010).

Differential weighting of substitution effects does not take into account other important information often available at no extra cost, such as genetic map positions of loci and linkage phases of alleles at different loci. New selection criteria utilizing this information could be defined based on the prospects that candidates produce superior gametes with favorable combinations of haplotypes. While the average performance of progeny of such candidates might be inferior to that of individuals selected on GEBVs alone, the top-performing individuals in the progeny are expected to be superior, which boosts the genetic gain achievable in future generations.

In this vein, Daetwyler *et al.* (2015) recently proposed a criterion called optimal haploid value (OHV), which aims at predicting the theoretically optimal combination of haplotypes in a gamete produced from a heterozygous candidate. The criterion was tested in simulations of a bread wheat breeding program and showed increased genetic gain compared to selection on GEBVs. In their study, genetic progress was measured as the performance of the best doubled haploid (DH) line (generated by chromosome doubling of a gamete) produced from selected individuals. By definition, OHV does not take into account the finite size of the breeding population; hence, it merely considers the possibility of a superior gamete, disregarding its probability (Han *et al.* 2017). Moreover, OHV requires the genome to be partitioned into haplotypes, and it is yet an unsolved problem how this should be optimally accomplished.

In view of these limitations, we herein propose a novel selection criterion called expected maximum haploid breeding value (EMBV). It characterizes the breeding potential of a candidate in terms of the performance of the top gametes it is able to produce. If a candidate is selected for recombination, it will contribute a certain number of gametes to the next generation. This number can be directly ascertained under controlled matings or easily estimated under random mating conditions. The EMBV is then defined as the expected GEBV of the best among all DH lines derived from these gametes. Hence, EMBV takes the finite population size into account and it is not necessary to partition the genome into haplotypes.

The objectives of our study were (i) to evaluate *in silico* the potential of EMBV as an alternative selection criterion in a generic recurrent selection (RS) program and (ii) compare it to the criteria OHV, wGEBV and GEBV with respect to genetic gain and genetic diversity across 50 selection cycles. The performance of OHV was assessed under optimal conditions with respect to the partitioning of the genome into haplotypes. In order to evaluate the effect of gene action on the comparison of the selection criteria, we compared purely additive gene action with completely dominant gene action at all loci. Furthermore, we considered the effect of population size, the number of selected individuals and the number of chromosomes on the relative performance of the different selection criteria.

## Material and Methods

### Genetic model

We considered a quantitative trait with additive and dominant gene action at all *L* loci. Each locus was bi-allelic with alleles $A_{l}$ and $B_{l}$ and possible unordered genotypes $A_{l}A_{l},$ $A_{l}B_{l}$ and $B_{l}B_{l}.$ We assumed that the locations of loci and of alleles on homologous chromosomes are known (phased genotypic data). For each locus of a diploid, heterozygous individual *i*, let $x_{il}^{\left(j\right)}\in \left\{0,1\right\}$ be an indicator variable indicating absence or presence of the $B_{l}$ allele at the *l*-th locus on the *j*-th haploid genome (j∈{1,2}, referring to the maternal and paternal genome). Then $x_{il}=x_{il}^{\left(1\right)}+x_{il}^{\left(2\right)}$ is a genotypic score counting the number of $B_{l}$ alleles. Following the genetic model of Lynch and Walsh (1998), we assumed that the genotypic value of an individual *i* with genotype $x_{il}$ at locus *l* is given by

$$G_{il}=\{\begin{array}{ll} 0 & \text{if}\;x_{\mathrm{il}}=0\\ \left(1+k_{\text{l}}\right)a_{\text{l}} & \text{if}\;x_{\mathrm{il}}=1\\ 2a_{\text{l}} & \text{if}\;x_{\mathrm{il}}=2 \end{array}$$ (1)

where $a_{l}$ is the homozygous effect (half the difference between the two homozygous genotypes) and $k_{l}$ the dominance coefficient (deviation of the heterozygote from the mean of the homozygotes in units of $a_{l}$). Effects $a_{l}$ were independently drawn from a gamma distribution Γ(shape=1.66,scale=0.4), following Meuwissen *et al.* (2001), such that $B_{l}$ was implicitly defined as the favorable allele. We assumed two extreme scenarios where gene action at all QTL was either purely additive, $k_{l}=0,$ or completely dominant/recessive, where $k_{l}$ was either 1 or −1, with equal probability. Dominance coefficients $k_{l}$ were assumed to be stochastically independent of additive effects, following Zeng *et al.* (2013). The genome-wide genotypic value of an individual *i* was computed as $G_{i}=\sum_{l}G_{i}.$ The average effect of an allelic substitution $\alpha_{l}$ at a single locus *l* was computed as $\alpha_{l}=a_{l}\left(1+k\left(1-2p_{l}\right)\right),$ where $p_{l}$ is the frequencies of allele $B_{l}$ at the respective locus. The BV of individual *i* was computed as $g_{i}=\sum_{l}\left(x_{il}-2p_{l}\right)\alpha_{l}$ (Vitezica *et al.* 2013). Following Daetwyler *et al.* (2015), we assumed that QTL genotypes and effects are known without error, *i.e.*, marker loci are identical to QTL and their associated allele substitution effects are identical to the simulated substitution effects for QTL. This was done in order to assess the performance of the investigated selection criteria under optimal conditions. Consequences in the practical case where the trait genetic architecture is unknown and (marker) allele substitution effects can be only estimated with some degree of precision are addressed in the discussion.

### Selection criteria

#### GEBV:

The GEBV of individual *i* is canonically computed as

$$GEBV_{i}=\sum_{l=1}^{L}x_{il}\alpha_{l},$$ (2)

which is the genome-wide sum of all substitution effects for the respective alleles.

#### EMBV:

**Figure 1:**
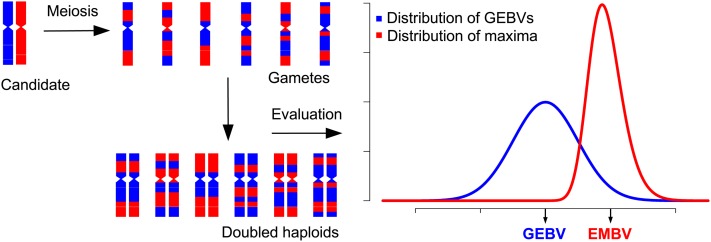
Illustration of the computation of EMBV for a heterozygous selection candidate. A (conceptually) infinite population of gametes is generated *in silico* from the candidate by simulating meiosis events. The corresponding doubled haploid (DH) lines are evaluated for their GEBVs, yielding a distribution of GEBVs (blue curve). The candidate’s GEBV corresponds to the mean GEBV of the DH lines. The EMBV is defined as the expected value of the maximum GEBV of a random sample of DH lines of size $N_{G},$ where $N_{G}$ is the expected number of gametes the candidate will contribute to the next generation.

The EMBV measures the breeding potential of a candidate in terms of the expected GEBV of the best out of $N_{G}$ DH lines produced by it (visualized in Figure 1), where $N_{G}$ denotes the number of gametes the candidate is expected to contribute to the next generation, if it is selected. If $Y_{i}$ denotes the GEBV of a random DH line produced by candidate *i*, the EMBV is formally defined as

$$EMBV_{i}=\text{E}\left(Y_{i\left(N_{G}\right)}\right),$$

where $Y_{i\left(N_{G}\right)}$ is the largest order statistic (maximum) of a random sample of size $N_{G}.$ An alternative formulation of EMBV using a normal approximation for the distribution of GEBVs of DH lines produced by *i* is provided in File S2 and discussed below.

#### OHV:

For the computation of OHV, the entire set of loci {1,…,L}, ordered along the genome, is partitioned into *N* disjoint non-empty subsets $S_{k}$ (corresponding to haplotypes), such that $\left\{1,\ldots ,L\right\}={\cup^{\text{​}}}_{k=1}^{N}S_{k}$ and $l<l^{\prime }$ for all $l\in S_{k},$ $l^{\prime }\in S_{k+1}$ and all 1≤k<N. According to Daetwyler *et al.* (2015), the OHV of a selection candidate *i* is computed as

$$OHV_{i}=2\sum_{k=1}^{N_{S}}\underset{j\in \left\{1,2\right\}}{\max}\left\{\underset{l\in S_{k}}{\sum }x_{il}^{\left(j\right)}\alpha_{l}\right\},$$ (3)

*i.e.*, for each haplotype, the maximum breeding value over all haploid genomes is determined and twice the sum of these values is taken as OHV.

#### wGEBV:

In selection criterion wGEBV marker effects are weighted by a coefficient that depends on the frequency $p_{l}$ of the favorable allele $B_{l}.$ The associated locus weights were computed according to Goddard (2009) as $\omega_{l}=\pi /2-\text{arcsin}\left(\sqrt{p_{l}}\right)/\sqrt{p_{l}\left(1-p_{l}\right)}$ and wGEBVs were calculated with the modification proposed by Jannink (2010) as

$$wGEBV_{i}=\sum_{l=1}^{L}x_{il}\omega_{l}\alpha_{l}.$$ (4)

For all criteria, allele frequency $p_{l}$ was freshly computed as the sample frequencies of allele $B_{l}$ in each cycle of the breeding program; accordingly, allele substitution effects $\alpha_{l}$ (and locus weights $\omega_{l}$ for wGEBV) varied between cycles. It is important to note that the EMBV and OHV of a completely homozygous individual are identical to its GEBV; hence, these selection criteria only differ for heterozygous genotypes. The computation of GEBV and wGEBV only requires genome-wide co-dominant bi-allelic markers with effect estimates, whereas both EMBV and OHV additionally require a genetic map and phased marker genotypes of the candidates. Criterion EMBV further requires software for simulating meiosis events (*e.g.*, Müller and Broman 2017).

### Simulation of the base population and genome structure

We considered a diploid species with a constant genome length of 2,000 cM. The genome was subdivided into $N_{chr}\in \left\{5,20,40\right\}$ segregating chromosomes with equal length (*i.e.*, 400, 100 and 50 cM, respectively). Bi-allelic QTL were uniformly distributed along the genome with a density of 2 QTL per cM, corresponding to a total of 1,000 QTL. The simulation of the base population was conducted as in Müller *et al.* (2017). Briefly, a historical population of 1,500 diploid individuals was subject to random mating for 3,000 generations. A population bottleneck was simulated by arbitrarily selecting 40 individuals that were further randomly mated for 15 generations to build up extensive linkage disequilibrium, as often observed in elite germplasm in plant breeding (*e.g.*, Van Inghelandt *et al.* 2011). The population was then expanded to 5,000 individuals and randomly mated for three more generations to remove close family relationships and establish the base population. Finally, all monomorphic loci were removed from the genotypic data. The base population was simulated only once for each value of $N_{chr}.$ The distribution of allele frequencies (data not shown) and linkage disequilibrium (Figure S1 in File S3) in terms of $r^{2}$ (Hill and Robertson 1968) was similar for different $N_{chr}.$

### Breeding program

From the base population, $N_{cand}$ individuals were randomly sampled without replacement and constituted the candidates in cycle $C_{0}.$ We considered four distinct breeding programs, starting from the same set of individuals in $C_{0},$ that only differed in the selection criterion, namely the use of GEBV, wGEBV, OHV or EMBV to select $N_{sel}$ candidates for establishing the next generation. For criterion EMBV, the number of gametes $N_{G}$ contributed, on average, by one selected individual to the next generation of $N_{cand}$ new candidates was estimated as $2N_{cand}/N_{sel},$ rounded to the nearest integer. In a given cycle $C_{t},$ all candidates were evaluated and ranked for the applied selection criterion and the best $N_{sel}$ candidates were selected. For creating cycle $C_{t+1},$ the selected individuals were randomly mated, *i.e.*, both parents of each future individual were randomly drawn with replacement from the selected candidates, allowing for self-fertilization (father = mother). One gamete per parent was produced and both gametes united to form the new progeny. In cycle $C_{0},$ the population mean (average of all genotypic values) and the standard deviation of BVs ($\sigma_{a_{0}}$) were calculated. In each later cycle $C_{t},$ all individuals were genotyped and the difference between the population mean in $C_{t}$ and $C_{0}$ was computed. This difference was then scaled by $\sigma_{a_{0}}$ and the result was recorded as the genetic gain (*R*), analogous to Jannink (2010). Hence, *R* is measured as the progress of the population mean in units of $\sigma_{a_{0}},$ relative to $C_{0}.$ Note that $\sigma_{a_{0}}$ can vary between the different scenarios and among samples of founder individuals from the base population within scenarios. Scaling by $\sigma_{a_{0}}$ aims to correct for this difference in the initially available additive variance, but does not affect comparisons between the four selection criteria. In each selection cycle *t*, genetic diversity was calculated as the variance of the BVs of all candidates ($\sigma_{a_{t}}^{2}$), divided by $\sigma_{a_{0}}^{2}.$ The breeding program was continued for a total of 50 selection cycles. The factors investigated in our simulations (Table 1) were (i) the number of candidates in each cycle, $N_{cand}\in \left\{30,50\right\},$ (ii) the number of selected individuals as parents for the next generation, $N_{sel}\in \left\{1,3,10\right\},$ (iii) the number of chromosomes, $N_{chr}\in \left\{5,20,40\right\},$ and (iv) the level of dominance, k=0 (no dominance) or k=±1 (complete dominance). The breeding program was replicated at least 600 times for each scenario, starting with sampling the initial candidates in $C_{0}$ from the base population and the simulation of homozygous effects and sampling of the signs of dominance coefficients. The homozygous effects were always scaled to achieve unit additive genetic variance in the base population. Summary statistics are generally reported as arithmetic means across all replicates.

**Table 1:** Factors investigated, with symbols and list of levels

Factor	Symbol	Levels
Number of selection candidates per generation	$N_{cand}$	30,50
Number of selected individuals per generation	$N_{sel}$	1,3,10
Number of non-homologous chromosome	$N_{chr}$	5,20,40
Mode of gene action	*k*	additive (k=0),
		dominant (k=±1)

Levels in boldface type identify the standard scenario.

### Computation of OHV and estimation of EMBV

The estimation of OHVs requires the specification of haplotypes. The most straightforward way, which we pursued, is to agree on a number of $N_{S}-1$ equidistant breakpoints that partition each chromosome into $N_{S}$ haplotypes of equal length (Daetwyler *et al.* 2015). We explored different values for $N_{S},$ starting from 1 (*i.e.*, entire chromosomes) and following the geometric sequence $2^{k},$ $k\in {\mathbb{N}}_{0},$ as long as the haplotypes had a length ≥6.25 cM. An overview of $N_{S}$ and segment lengths for different $N_{chr}$ is shown in Table S1-1 in File S1 and results for *R* obtained with criterion OHV are described in File S1. In the following, we only show those results for criterion OHV where $N_{S}$ was found to yield maximum *R* after 50 cycles of selection.

While GEBVs, OHVs and wGEBVs can be directly computed from genotypic data and allele substitution effects, the estimation of EMBVs is computationally demanding, because an overall large number of DHs has to be generated per individual. We estimated EMBVs by repeatedly producing $N_{G}$ gametes, determining the maximum GEBV among them as described above, and taking the arithmetic mean of the maxima over all replicates. The number of replicates was dynamically adapted such that the empirical standard error was smaller than 0.01 (but at least 10 replicates were taken). This strategy was chosen to balance estimation accuracy and computation time, but in practical applications, computation time is not a bottleneck. We developed a C++ routine for the fast estimation of EMBVs, which is publicly available via a wrapper R package *embvr* (Müller 2017). A possible alternative approach for rapid analytical computation of EMBVs is described in File S2.

### Data availability

Datasets and source code used in our simulations are publicly available from https://doi.org/10.5281/zenodo.1161723. File supplental_figures contains supplementary figures. File supplement_1 contains results on the optimal number of haplotypes for selection criterion OHV. File supplement_2 presents an approximation of EMBV using the normal distribution.

## Results

**Figure 2:**
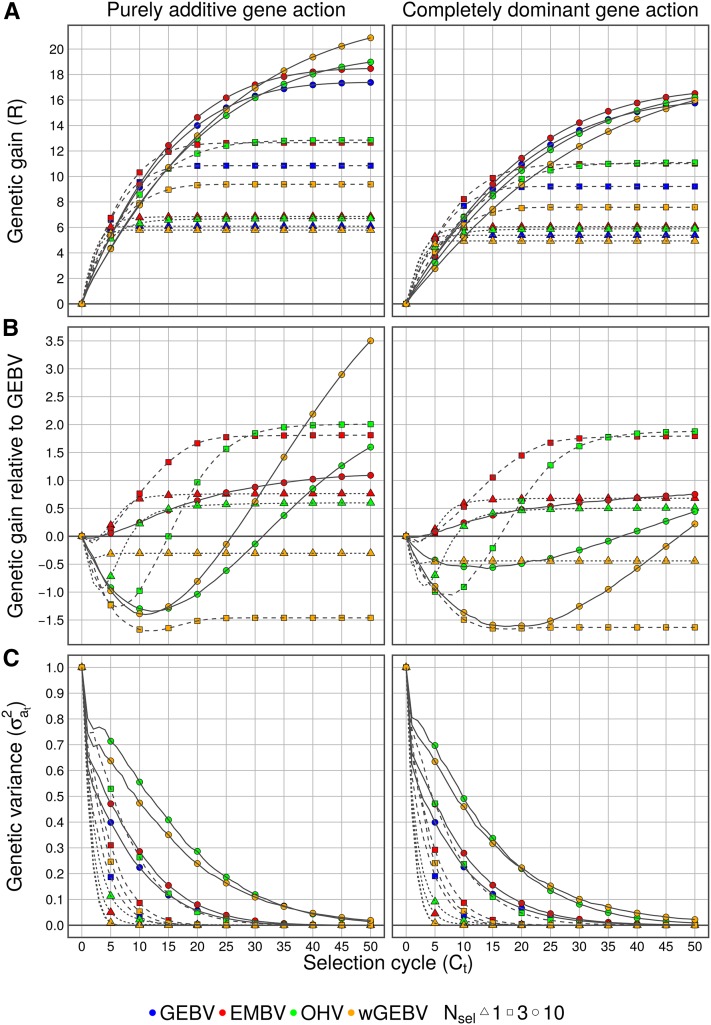
(A) Genetic gain (*R*), (B) relative genetic gain and (C) additive variance ($\sigma_{a_{t}}^{2}$) for selection criteria genomic-estimated breeding value (GEBV), expected maximum haploid breeding value (EMBV), optimal haploid value (OHV) and weighted GEBV (wGEBV) under recurrent selection. Results refer to $N_{chr}=20$ and $N_{cand}=50.$ $N_{chr},$ number of chromosomes; $N_{cand},$ number of selection candidates; $N_{sel},$ number of selected individuals.

The genetic gain *R* generally approached a plateau (selection limit) for all selection criteria as the breeding program proceeded (Figure 2a). During selection, an increasing number of causal polymorphisms became fixed, such that in late stages of the breeding program, individuals were nearly homozygous and the genetic variance was depleted (Figure 2c). An exception was selection criterion wGEBV, where still considerable genetic progress was achieved after 50 cycles of selection. The rate of genetic progress and the selection limit depended on the selection pressure via the number of selected individuals $N_{sel}.$ If only a single candidate was selected ($N_{sel}=1$), which corresponds to recurrent selfing, genetic progress was initially very fast, but *R* quickly reached a low selection limit after about 10 cycles. Conversely, under mild selection pressure with $N_{sel}=10,$ genetic progress was slow at the beginning, but endured over the entire breeding program and *R* generally did not fully reach the selection limit, even after 50 cycles.

### Genetic gain

#### Additive gene action:

Selection criterion EMBV was, in advanced selection cycles, clearly superior to GEBV in terms of genetic gain (Figure 2a), but minimally weaker in the first cycles (until about cycle 5). After this point, $R_{EMBV}$ surpassed and strictly increased relative to $R_{GEBV}$ during selection. After 50 cycles, $R_{EMBV}$ reached a genetic gain of 12.5% ($N_{sel}=1$), 16.7% ($N_{sel}=3$) and 6.3% ($N_{sel}=10$) larger than $R_{GEBV}.$ With selection criterion OHV, $R_{OHV}$ increased at a lower rate than $R_{GEBV}$ in early cycles. However, $R_{OHV}$ generally caught up to $R_{GEBV}$ and eventually surpassed it. The larger $N_{sel},$ the more cycles it took for $R_{OHV}$ to surpass $R_{GEBV}$ (9 cycles for $N_{sel}=1,$ compared to 38 cycles for $N_{sel}=10$). After 50 cycles, $R_{OHV}$ was 9.8% higher than $R_{GEBV}$ for $N_{sel}=1,$ but 18.5% and 9.2% higher for $N_{sel}=3$ and 10, respectively, exceeding the performance of EMBV. Criterion wGEBV showed a unique behavior. In general, $R_{wGEBV}$ increased slower than $R_{GEBV}$ in the first few cycles, similar to OHV, and plateaued for $N_{sel}=1$ and 3 at a level −5% and −13.5%, respectively, below $R_{GEBV}.$ However, for $N_{sel}=10,$ although $R_{wGEBV}$ also initially slowly increased, it surpassed $R_{GEBV}$ after 25 cycles and eventually reached a value 20.15% larger than $R_{GEBV}$ after 50 cycles, also surmounting all other criteria.

#### Dominant gene action:

If gene action at all loci was completely dominant, both the overall level of *R* (Figure 2a), as well as the advantage of the alternative selection criteria over GEBV (Figure 2b) were reduced, but the extent depended on the criterion. While EMBV appeared to be robust to dominant gene action for different values of $N_{sel},$ $R_{OHV}$ and $R_{wGEBV}$ were severely reduced for $N_{sel}=10,$ reaching only 2.8% ($R_{OHV}$) and 1.4% ($R_{wGEBV}$) more than $R_{GEBV}$ after 50 cycles.

#### Number of candidates and chromosomes:

**Figure 3:**
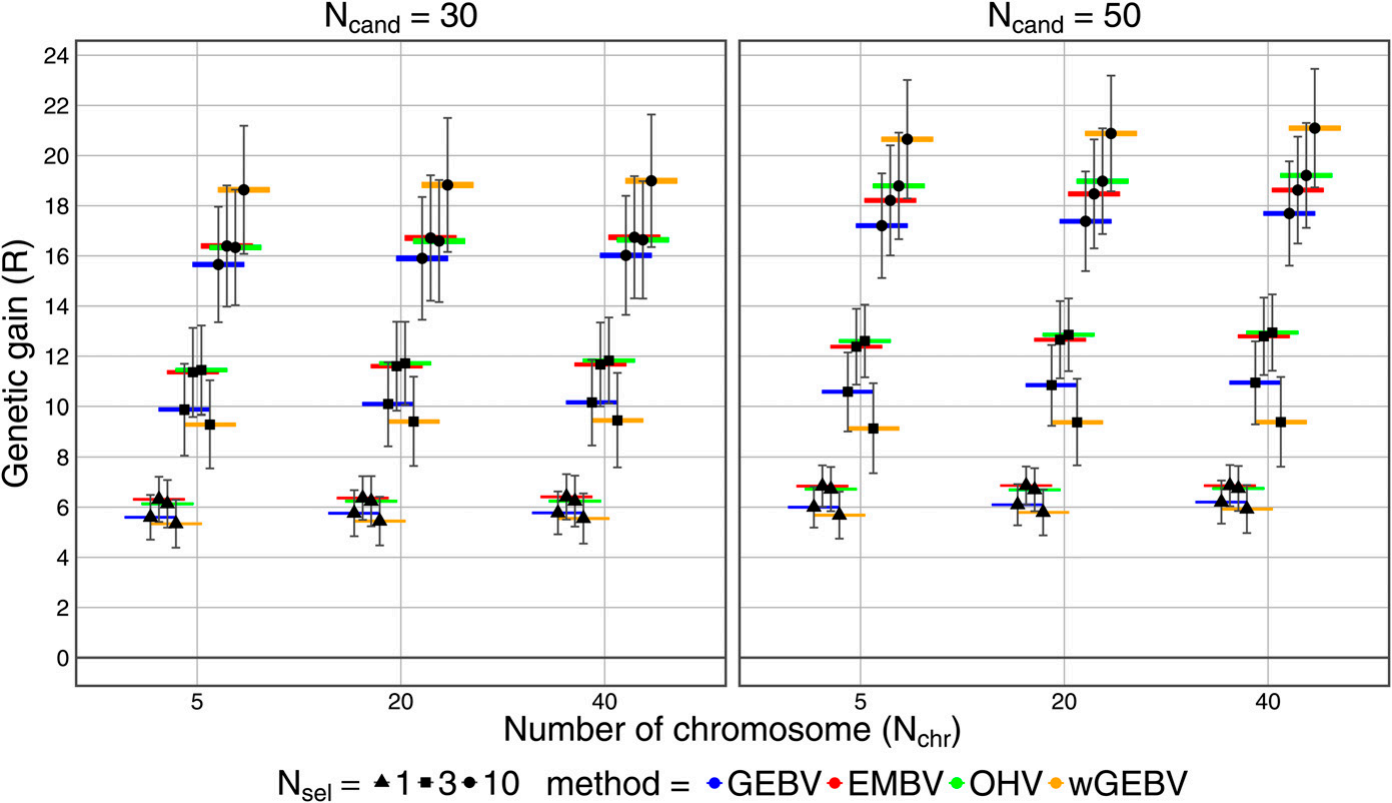
Genetic gain (*R*) in cycle $C_{50}$ for selection criteria genomic-estimated breeding value (GEBV), expected maximum haploid breeding value (EMBV), optimal haploid value (OHV) and weighted GEBV (wGEBV) under recurrent selection with purely additive gene action. Boxes and whiskers indicate standard errors and standard deviations across replicates, respectively. $N_{chr},$ number of chromosomes; $N_{cand},$ number of selection candidates; $N_{sel},$ number of selected individuals.

Reducing the number of selection candidates $N_{cand}$ from 50 (standard scenario) to 30 lead to a reduction in the overall level of *R* for all selection criteria (Figure 3). The larger population size with $N_{cand}=50$ caused a slightly higher allelic diversity in $C_{0},$ calculated as the average number of alleles per QTL, of 1.97 compared to 1.94 for $N_{cand}=30.$ This increases the probability that rare favorable alleles in the base population are also present in the breeding population, and hence benefits long-term genetic gain. The ranking between different selection criteria for $N_{cand}=30$ was similar to $N_{cand}=50.$ Comparing OHV with EMBV, $R_{OHV}$ tended to decrease relative to $R_{EMBV},$ when $N_{cand}$ was lowered from 50 to 30 individuals.

Larger $N_{chr}$ slightly elevated the overall level of *R* for all selection criteria (Figure S2 in File S3). With a constant genome size of 2,000 cM assumed in our study, increasing $N_{chr}$ increased the overall number of recombinations between loci, which benefited long-term genetic gain. The relative differences in *R* gain between the selection criteria was hardly influenced by $N_{chr}.$ However, it must be taken into account that for OHV, we considered only the optimal number of haplotypes $N_{S}.$ For instance, choosing $N_{S}=2$ per chromosome yielded optimal *R* only for $N_{chr}=40,$ but not for $N_{chr}=5$ (Figure S1-3 in File S1).

### Genetic diversity

The criteria EMBV and OHV generally showed the ability to maintain higher genetic diversity in terms of $\sigma_{a_{t}}^{2}$ in the population than GEBV, while criterion wGEBV only showed larger $\sigma_{a_{t}}^{2}$ than GEBV for $N_{sel}=10$ (Figure 2c). The rate of decline of $\sigma_{a_{t}}^{2}$ became more pronounced when $N_{sel}$ was reduced from 10 to 1. Across all cycles, $\sigma_{a_{t}}^{2}$ was always larger for criterion OHV compared to GEBV. After 50 cycles, $\sigma_{a_{t}}^{2}$ was entirely depleted with EMBV and GEBV, but not with OHV and wGEBV for $N_{sel}=10.$ Here, 1.4% (OHV) and 1.9% (wGEBV) of $\sigma_{a_{0}}^{2}$ was left. For $N_{cand}=30,$ wGEBV showed a higher $\sigma_{a_{t}}^{2}$ of 4.1% in cycle 50 that for $N_{cand}=50$ (Figure S6 in File S3). Remnant $\sigma_{a_{t}}^{2}$ explains why the selection limit was not fully reached in the case of OHV and wGEBV (Figure 2a). This indicates that the final genetic gain of OHV and wGEBV would have been higher if selection was continued for more than 50 cycles. Generally, $N_{chr}$ and $N_{cand}$ had only small effects on $\sigma_{a_{t}}^{2}$ for the different selection criteria (Figure S6 in File S3). Trends were similar under completely dominant gene action (Figure 2c, Figure S7 in File S3).

## Discussion

Genomic selection allows for predicting GEBVs of unphenotyped individuals and has been proposed for RS to increase genetic gain per unit time (Windhausen *et al.* 2012; Gorjanc *et al.* 2016). A first empirical study on GS in a multi-parental population produced from 18 tropical maize lines showed promising results, reporting 2% genetic gain in grain yield per year (Zhang *et al.* 2017). However, selection on GEBVs is expected to maximize single-cycle genetic gain, but not genetic gain over several cycles. In this study, we propose a novel selection criterion called expected maximum haploid breeding value (EMBV) as an alternative to the use of GEBVs for RS. EMBV takes into account information about genetic map positions of loci, linkage phases between alleles and the population size to improved long-term genetic gain. We used extensive computer simulations to compare EMBV to two other alternative selection criteria, wGEBV and OHV (Goddard 2009; Jannink 2010; Daetwyler *et al.* 2015) in a generic RS program.

RS was pioneered in maize (*Zea mays* L.) breeding (Jenkins 1940; Hull 1945; Comstock *et al.* 1949) and two basic types of selection strategies have been developed, intra- and inter-population improvement, where the latter is also called reciprocal RS. RS had only a limited but yet significant impact on the development of improved inbred lines in commercial hybrid breeding. Most notably, the Iowa Stiff Stalk Synthetic produced many successful inbred lines and its traces are present in a large proportion of today’s elite germplasm (Mikel and Dudley 2006; Hallauer and Carena 2012). Because of the historically limited success of RS, Hallauer and Carena (2012) recommend to tightly integrate the development of elite inbreed lines with germplasm enhancement programs driven by RS. This is particularly facilitated by the DH technology, which allows for rapid development of fully homozygous lines ready for testcross evaluation. While RS (either intra- or inter-population) can be used to steadily improve the germplasm, DH lines can be simultaneously created and tested as spin-offs from top parents. We expect EMBV to be also highly suitable for the selection of such DH parents, because by its very definition, it enables the identification of parents that most likely produce top performing DH lines. Genetic progress is then not measured in terms of population mean performance, but in terms of the performance of the best DH that can be achieved for line development, similar to Daetwyler *et al.* (2015). If EMBV is deployed for both RS and spin-off DH production, the parents used for DH line development do not need to be recruited from the individuals selected for intercrossing, but can constitute a separate set. This is because the ranking of the candidates in both applications will likely differ due to (i) differences in $N_{G}$ and (ii) differences in allele substitution effect estimates, which occur if different testers are used and gene action is not purely additive. For intra-population RS, the tester is the (current) population (*e.g.*, evaluation of half-sibs), whereas for inter-population RS, the tester stems from the opposite heterotic group. In both cases, the selection of DH parents requires substitution effects being estimated from testcrosses. EMBV might also be successfully applied independently of RS in advanced hybrid breeding programs, where new lines are commonly developed from bi-parental crosses between recycled elite lines. However, these extensions require further investigation.

### EMBV

The EMBV is an independent property of each selection candidate and is derived from the distribution of their virtual DH progeny. By this approach, the ultimate goal of using EMBVs is not to maximize genetic gain in the subsequent generation, but to improve gain in later stages of the breeding program. This is underlined by our result that selection on EMBVs needed around 5 cycles to outperform GEBV (Figures S4 and S8 in File S3), even though the initial penalty of using EMBVs was minimal. By selection on EMBVs, only individuals that are expected to produce the best gametes in the next generation are advanced. If such top gametes eventually unite, a superior individual is created, which, if selected for further breeding, can increase the population mean of future selection cycles. Due to the linearity of expectations, the EMBV can also be expressed as

$$EMBV_{i}=GEBV_{i}+E\left(X_{\left(N_{G}\right)}\right)\sigma_{i},$$ (5)

where $GEBV_{i}$ is the GEBV of candidate *i*, $\sigma_{i}$ is the standard deviation of the GEBVs of the DH lines derived from *i*, and $E\left(X_{\left(N_{G}\right)}\right)$ the expected value of the largest order statistic of $N_{G}$ random variables from N(0,1), assuming the GEBVs of the virtual DH progeny are normally distributed. This is described in greater detail in File S2. When expressed in this way, the EMBV can be immediately interpreted as a compromise between the candidate’s GEBV (current breeding potential) and its segregation variance (indicative of future breeding potential). Increasing the number of contributed gametes $N_{G}$ increases $E\left(X_{\left(N_{G}\right)}\right)$ (Figure S2-1 in File S2and hence the importance of $\sigma_{i}.$ Hence, the ranking of candidates can vary, depending on $N_{G}$ (see Figure S2-3 in File S2 for an example). Candidates with intermediate GEBVs showed larger variation in $\sigma_{i}$ compared to candidates with low or high GEBVs. Therefore, selection on EMBV often times chooses candidates with suboptimal GEBV, but in return larger $\sigma_{i}.$

### OHV

Application of criterion OHV requires the definition of haplotypes, from which the optimal combination of haplotype values is calculated. OHV conceptually fits into the framework of EMBV in that $EMBV_{i}\to OHV_{i}$ for $N_{G}\to \infty ,$ given complete linkage among loci within a haplotype but free recombination between haplotypes. The need for an explicit specification of haplotypes could be considered as a disadvantage of OHV. Our results demonstrate that OHV has a large potential to boost long-term genetic gain. However, these results might be overly optimistic, because we only used optimal values of $N_{S}.$ As Daetwyler *et al.* (2015) pointed out, decreasing $N_{S}$ (increasing haplotype lengths) shifts the breeding goal of maximizing genetic gain into the future, which is underlined by our results (Figure S1-1 in File S1). The reason is that a gamete exhibiting the OHV (or being at least close to it) can only be produced through the accumulation of favorable recombination events close to the haplotype borders. By definition, OHV only considers the possibility of the optimal gamete combining only the best haplotypes, not taking into account its probability of occurrence (Han *et al.* 2017). If $N_{S}$ is chosen such that genetic gain is maximized at an earlier stage of the breeding program, gain in cycle 50 is compromised (results not shown). As a consequence, OHV needs to be tuned according to the length of the breeding program. We observed substantial losses of genetic gain for OHV relative to GEBV in early selection cycles, in accordance with a simulation study by Goiffon *et al.* (2017). This was not found by Daetwyler *et al.* (2015), likely because they evaluated genetic progress in terms of the performance of only the best DH produced from all selected individuals. Hence, if a (nearly) optimal gamete is eventually produced, it will directly and exclusively enter into the measurement of genetic gain. Conversely, we measured genetic progress as the average genotypic value of the entire breeding population.

### wGEBV

Criterion wGEBV was unique because it performed poorly for small $N_{sel},$ but clearly outperformed all other criteria for $N_{sel}=10$ in terms of long-term genetic gain. In the latter case, remnant $\sigma_{a_{t}}^{2}$ suggests that if selection was continued further, the difference would have been even larger. We suspect that wGEBV is not competitive for small $N_{sel}$ because of strong genetic drift. This will rapidly result in a loss of many highly favorable but low-frequency alleles from the population. Only a very limited number of recombination events occur before individuals ultimately become homozygous; hence, there is not enough opportunity for combinations of favorable alleles to appear. Below, we explain why we expect that the superiority of wGEBV for $N_{sel}=10$ is likely overestimated.

Recently, Liu *et al.* (2015) proposed a further modification of the original approach to wGEBV. In their study, the effect weights are not only determined by the favorable allele frequency and change due to shifts in the latter, but also by a parameter regulating the initial weight at the beginning of selection and by the number of remaining generations until the end of the breeding program (“time horizon”). The closer the breeding program comes to its end, the lower the weight on effects with low favorable allele frequencies. They showed that their modified approach can improve on wGEBV in terms of long-term genetic gain. However, similar to wGEBV in our study, their method showed a clear performance penalty during the first cycles.

### Genetic diversity

The genetic diversity maintained in the breeding population was substantially higher for EMBV than GEBV. Selection based on GEBVs puts rare favorable alleles at a high risk of becoming lost. This is because such alleles will occur only in a small number of candidates. If they coincide with many unfavorable alleles, their positive effect is concealed. In other words, if rare favorable alleles are only present in candidates with an otherwise low GEBV, they will likely be lost. On the other hand, criterion EMBV allows rare favorable alleles to recombine and be joined with other favorable alleles into a high-performing gamete, reducing negative selection pressure on them. Moreover, the interpretation of the EMBV as a compromise between the GEBV and the segregation variance makes it evident that EMBV positively weights and maintains diversity. Criterion OHV maintained the highest diversity. Because it is computed as the sum of only the favorable haplotypes, it allows rare favorable alleles, similar to EMBV, to be separated from unfavorable alleles on other haplotypes and joined with favorable ones. Similar to OHV, criterion wGEBV was able to maintain relatively high genetic diversity, but only for $N_{sel}=10.$ This is because the differential weighting leads to a strong selection of rare favorable alleles (Jannink 2010). This effect was canceled by genetic drift if $N_{sel}$ was small.

### Effect of dominant gene action

In inter-population RS, breeders usually apply the same or closely related testers from the opposite heteroric group for several selection cycles. In this case, the genetic model for testcross performance behaves (in the absence of epistasis) like a model with only additive gene action (Melchinger *et al.* 1998), so that our simulations assuming additive gene action closely reflect this situation. However, for intra-population RS, the current cycle usually serves as a tester, and therefore allele frequencies are variable. Thus, in the presence of dominant gene action ($k_{l}\neq 0$) the allele substitution effects will change with changes in the allele frequencies across selection cycles. Moreover, in reality dominant gene action appears to be the rule rather than the exception (Crnokrak and Roff 1995; Hill *et al.* 2008). For these reasons, we investigated the extreme case of completely dominant gene action at all loci to assess the potential impact on the comparison of the selection criteria. Our results showed that EMBV and, to a lesser extent OHV, are robust with respect to dominance. On the other hand, the performance of wGEBV was severely affected under complete dominance. An explanation for the good performance of EMBV and OHV could be that these criteria are based on the assessment of homozygous individuals (DH lines), which removes the masking effect on recessive alleles present in heterozygotes, which affects criteria GEBV and wGEBV. Moreover, wGEBV was specifically proposed as a criterion for long-term population improvement, so it is implicitly assumed that substitution effects have a long-lasting significance, which does not hold under dominant gene action if allele frequencies change.

### Further research

#### Estimation of allele substitution effects:

In our study, we assumed that both the loci as well as the effect sizes of QTL are perfectly known. In practice, QTL are generally unknown and markers are used as proxies and their allele substitution effects have to be estimated from a training set, using one of several available analytical methods (*cf*. de los Campos *et al.* 2013). However, a high degree of colinearity among markers, especially in high density marker panels, entails that the effect of a QTL is distributed among surrounding markers in a complex manner, reducing the statistical power to accurately estimate their effects (Liu *et al.* 2015; Chang *et al.* 2018) Obviously, the accuracy of marker effects is further impaired for traits with low heritability or if insufficient phenotypic data are available.

We expect that estimation of allele substitution effects of markers will differently affect the selection criteria. In wGEBV, individual effects are weighted by the frequency of the favorable allele. However, the more inaccurate marker effects are estimated, the lower the significance of their effects and the higher the chance that the wrong allele is considered being favorable, potentially causing selection in the wrong direction. We therefore surmise that the potential of wGEBV is largely overestimated by the assumption of known substitution effects. Conversely, the criteria OHV and EMBV do not rely on individual marker effects, but consider entire haplotypes. This is done explicitly in OHV and implicitly in EMBV, because the virtual DH progeny of a selection candidate reflect colinearity among markers due to cosegregation. Hence, we expect that these criteria are less susceptible to inaccuracies in effect estimates, but this warrants further research.

Furthermore, as the predictive information of effect estimates erodes over multiple selection cycles due to changes in linkage disequilibrium between QTL and markers (*e.g.*, Muir 2007; Müller *et al.* 2017), periodic re-training of the prediction model is required, for instance every third cycle (Jannink 2010). Substitution effects should also be recalculated in every generation based on the allele frequencies of the respective tester, which is especially relevant if only a small fraction of the candidates is advanced.

#### Phasing:

The application of selection criteria EMBV and OHV requires the availability of phased genotypic data. If selection candidates are F1 crosses from homozygous inbred lines, all linkage phases are known. Otherwise, genotypes have to be phased before the candidates can be evaluated. In the past years, numerous software tools have been developed to achieve this task, *e.g.*, PHASE (Stephens *et al.* 2001), its successor fastPHASE (Scheet and Stephens 2006) or BEAGLE (Browning and Browning 2007). However, as phasing is still associated with a certain error rate, additional investigations are required to assess the influence of phasing error on the performance of EMBV and OHV. While selection criteria GEBV and wGEBV will stay unaffected by phasing errors, we expect that EMBV and OHV could both show a slightly reduced performance.

### Conclusions

We showed in a proof-of-concept that our novel selection criterion EMBV has the potential to yield higher long-term genetic gain as compared to using GEBVs while not jeopardizing short-term gain. Although criterion OHV performed well in the long run, it was not competitive with GEBV in early cycles, which makes it unattractive for practical breeding programs. Criterion wGEBV also showed promising long-term results for quantitative traits with purely additive gene action, but was also accompanied by a performance penalty in early cycles and was, moreover, sensitive to deviation from additive gene action. EMBV could also be a promising approach for the selection of parents for producing DH lines in hybrid breeding programs, which is a subject of future research.

## Supplementary Material

Supplemental Material is available online at www.g3journal.org/lookup/suppl/doi:10.1534/g3.118.200091/-/DC1
