# Supplementary material for: Selection on Expected Maximum Haploid Breeding Values Can Increase Genetic Gain in Recurrent Genomic Selection
Source: G3 (Bethesda). 2018 Feb 6;8(4):1173–81. doi: 10.1534/g3.118.200091 (PMC5873908; doi:10.1534/g3.118.200091)
Supplement: Supplementary file 1 [file 1173FileS1.pdf]

Selection on expected maximum haploid breeding values can increase genetic gain in recurrent genomic selection

Dominik Müller*, Pascal Schopp* and Albrecht E. Melchinger*

*Institute of Plant Breeding, Seed Sciences and Population Genetics, University of Hohenheim, 70599 Stuttgart, Germany

January 28th, 2018

Correspondence: melchinger@uni-hohenheim.de

Supplemental File 1

Optimal number of haplotypes for OHV

The optimal number of haplotypes N_S per chromosome for selection criterion OHV that maximized R_{OHV} in selection cycle 50 primarily depended on N_{chr} (Figure S1-3). For $N_{chr} = 5$ the optimal N_S ranged between 8 and 16. For $N_{chr} = 20$, it ranged between 2 and 4, and for $N_{chr} = 40$ between 1 and 2. Given the chromosomes lengths for these values of N_{chr} (400, 100 and 50 cM, respectively), the results indicate that the optimal haplotype length for criterion OHV that maximized long-term genetic gain after 50 cycles of selection corresponded to 25 or 50 cM (*cf.* Table S1-1). The number of selected individuals N_{sel} had no influence on N_S. The influence of the size of the breeding population N_{cand} was also relative small and a clear difference was only discernible for $N_{chr} = 20$. Here, the optimal N_S tended to be smaller with $N_S = 2$ for $N_{cand} = 30$ compared to $N_S = 4$ for $N_{cand} = 50$. The optimal N_S was different if the goal was to maximize R_{OHV} in earlier selection cycles (Figure S1-1). Here, our results indicate that the optimal N_S increased with the number of cycles, whereas it decreased with N_{sel}. With completely dominant gene action, N_S tended to be

smaller for $N_{cand} = 30$ by about a factor of two (Figure S1-2). For $N_{cand} = 50$, it was only smaller when $N_{sel} = 10$.

Tables

Table S1-1: Number of haplotypes (N_S) per chromosome for selection criterion OHV. Bold face numbers indicate the optimal N_S maximizing genetic gain R_{OHV} after 50 cycles of recurrent selection for different values of N_{cand} and N_{sel}.

N_{chr}	L	length (cM)						
		6.25	12.5	25	50	100	200	400
5	400	64	32	**16**	**8**	4	2	1
20	100	16	8	**4**	**2**	1	-	-
40	50	8	4	**2**	**1**	-	-	-

N_{chr}, number of chromosomes; L, chromosome length; N_{cand}, number of selection candidates; N_{sel}, number of selected individuals

Figures

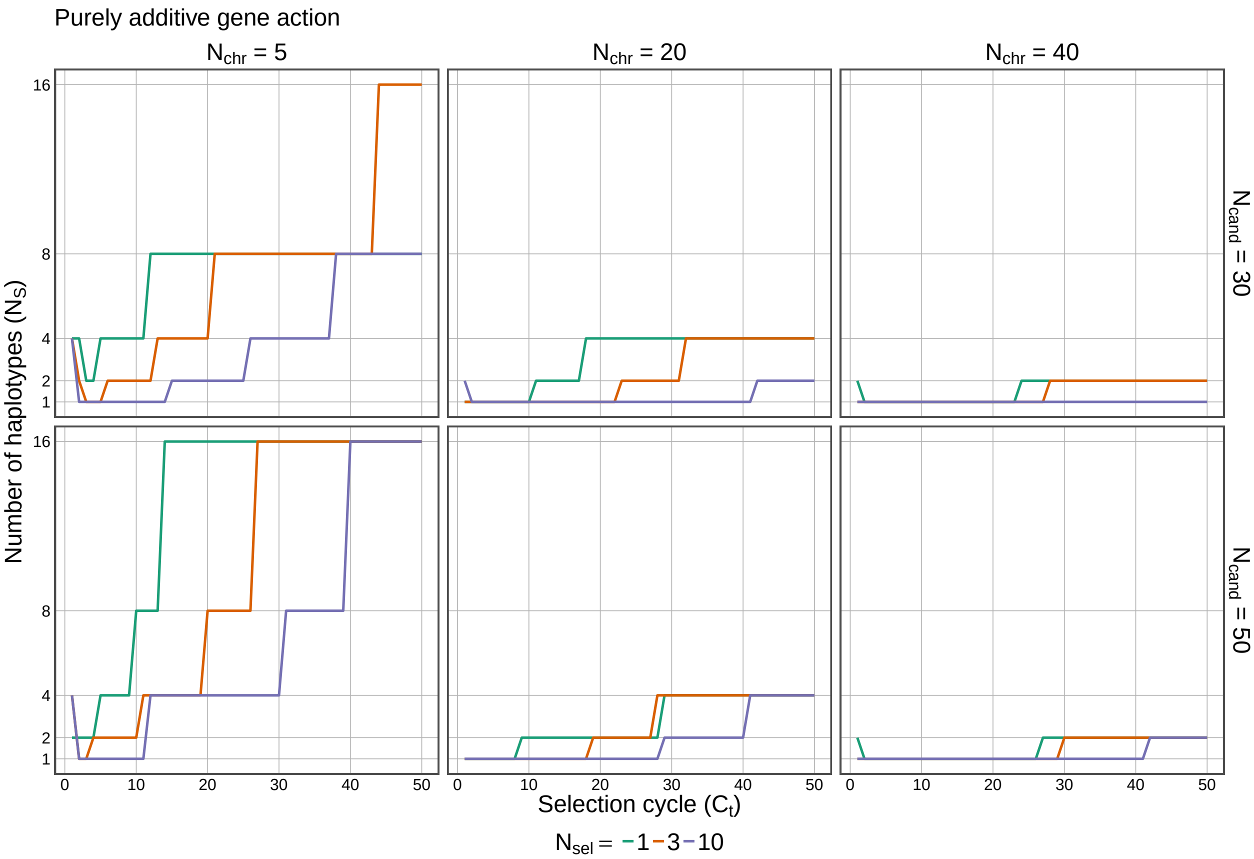

Figure S1-1: Number of haplotypes N_S per chromosome that maximize genetic gain (R_{OHV}) in the respective selection cycle for scenario optimal haploid value (OHV) under purely additive gene action. N_{chr}, number of chromosomes; N_{cand}, number of selection candidates; N_{sel}, number of selected individuals.

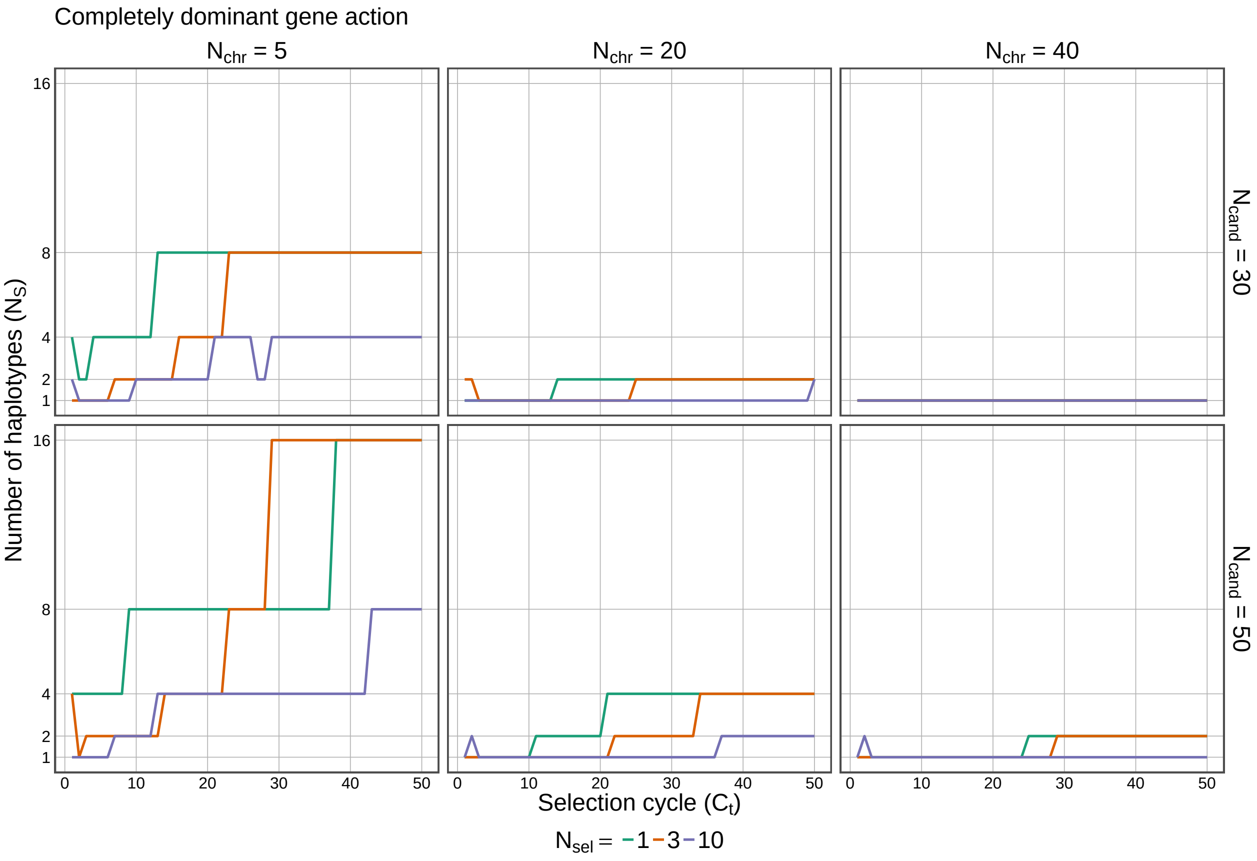

Figure S1-2: Number of haplotypes N_S per chromosome that maximize genetic gain (R_{OHV}) in the respective selection cycle for scenario optimal haploid value (OHV) under completely dominant gene action. N_{chr}, number of chromosomes; N_{cand}, number of selection candidates; N_{sel}, number of selected individuals.

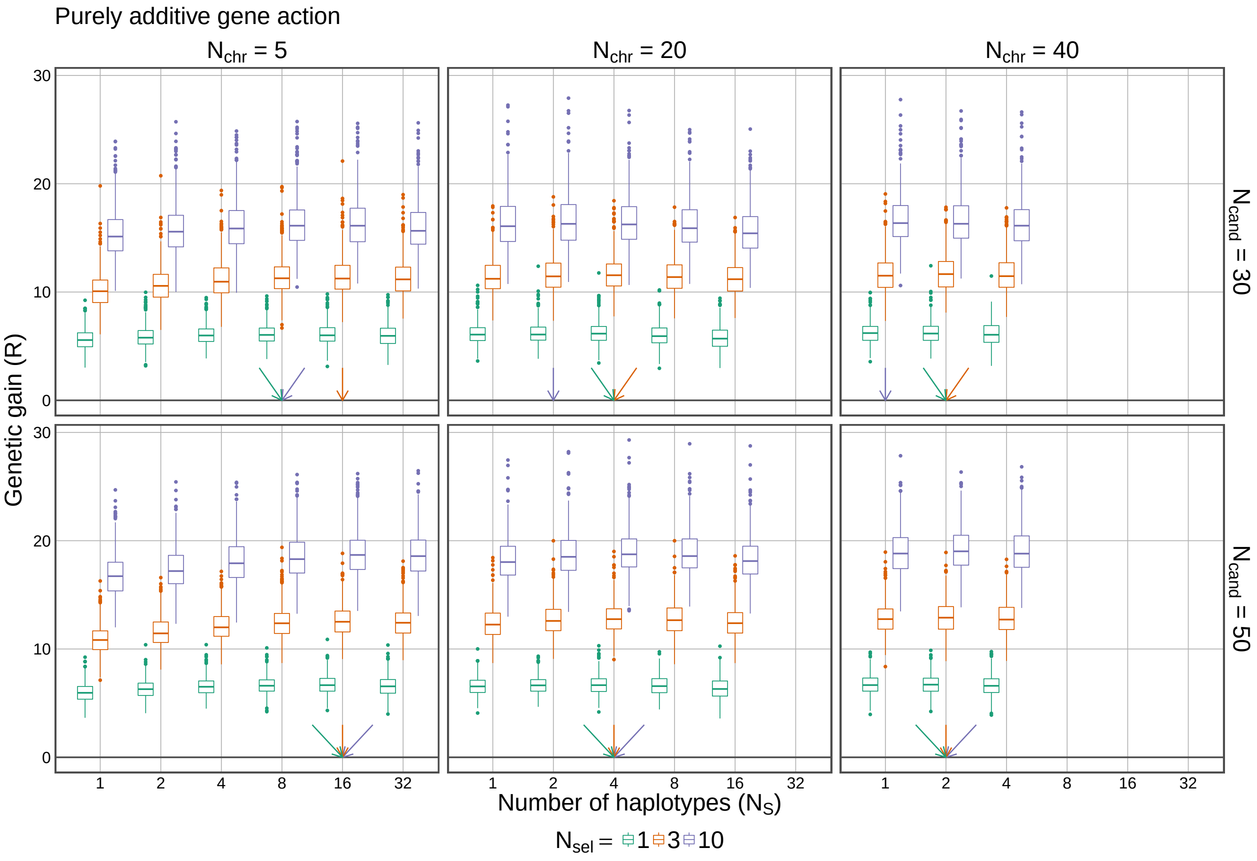

Figure S1-3: Boxplots of genetic gain (R_{OHV}) for selection criterion optimal haploid value (OHV) in selection cycle 50 under purely additive gene action. N_{chr}, number of chromosomes; N_{cand}, number of selection candidates; N_{sel}, number of selected individuals; N_S, number of haplotypes per chromosome. Arrows indicate the optimal number of N_S.

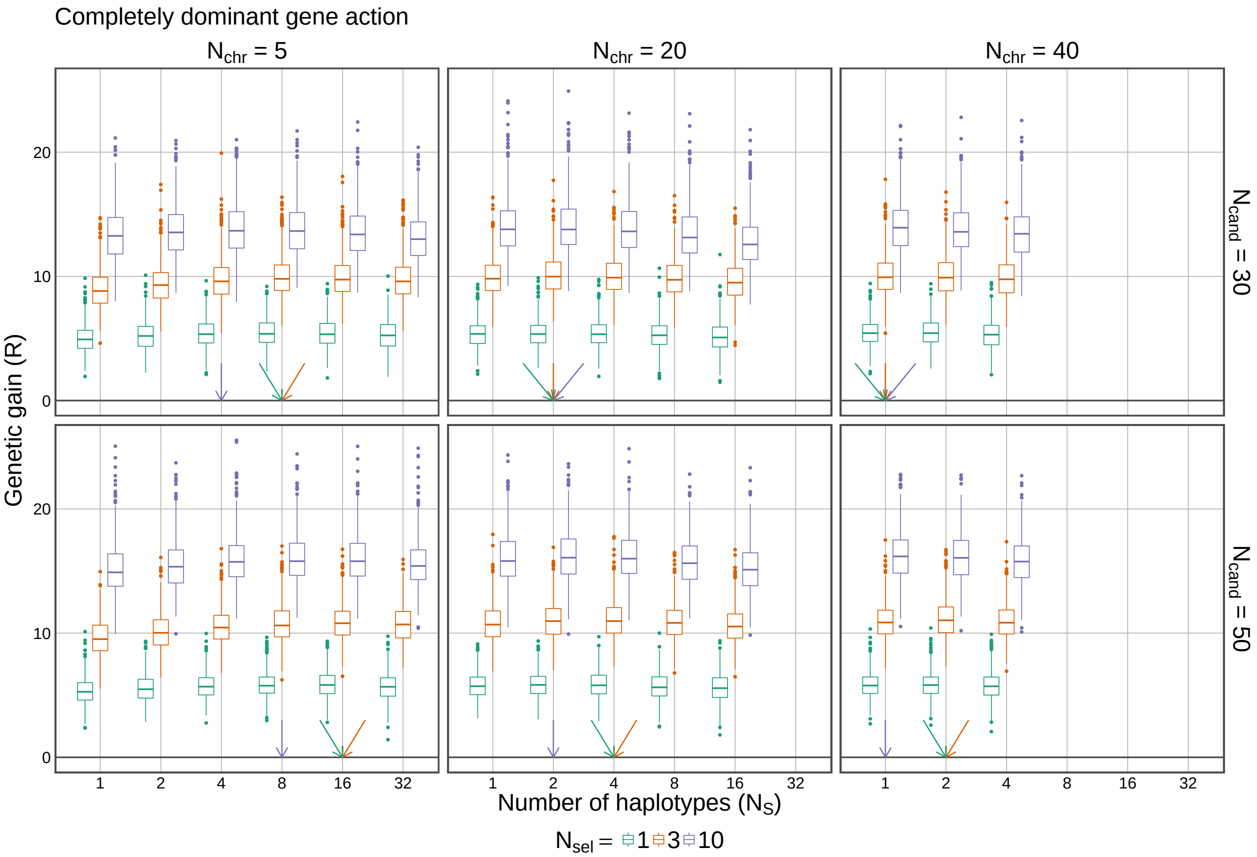

Figure S1-4: Boxplots of genetic gain (R_{OHV}) for selection criterion optimal haploid value (OHV) in selection cycle 50 under completely dominant gene action. N_{chr}, number of chromosomes; N_{cand}, number of selection candidates; N_{sel}, number of selected individuals; N_S, number of haplotypes per chromosome. Arrows indicate the optimal number of N_S.
