# Supplementary material for: Selection on Expected Maximum Haploid Breeding Values Can Increase Genetic Gain in Recurrent Genomic Selection
Source: G3 (Bethesda). 2018 Feb 6;8(4):1173–81. doi: 10.1534/g3.118.200091 (PMC5873908; doi:10.1534/g3.118.200091)
Supplement: Supplementary file 2 [file 1173FileS2.pdf]

Selection on expected maximum haploid breeding values can increase genetic gain in recurrent genomic selection

Dominik Müller∗, Pascal Schopp∗ and Albrecht E. Melchinger∗

∗Institute of Plant Breeding, Seed Sciences and Population Genetics, University of Hohenheim, 70599 Stuttgart, Germany

January 28th, 2018

Correspondence: melchinger@uni-hohenheim.de

Supplemental File 2

Approximation of EMBV using a normal distribution

Using independent assortment of chromosomes and conclusions following from the central limit theorem, breeding values of a quantitative trait asymptotically follow a normal distribution. Let σ_i^2 be the segregation variance in the progeny of a candidate i, defined as the variance of the breeding values of a population of DH lines developed from it. Let $GEBV_i$ be the GEBV of the candidate. Then, for the breeding values of these DH lines, we have $Y_i \sim \mathcal{N}(GEBV_i, \sigma_i^2)$. If we denote with $Y_{i(N_G)}$ the largest order statistic (maximum) of a sample of N_G DH lines, the definition of the EMBV implies that $EMBV = E\left(Y_{(N_G)}\right)$. By the properties of the expectation and of order statistics, it is easy to see that

$$EMBV_i = GEBV_i + E\left(X_{(N_G)}\right)\sigma_i, \tag{1}$$

where $E\left(X_{(N_G)}\right)$ is the expectation of the largest order statistic of N_G random variables from $\mathcal{N}(0, 1)$ (Figure S2-1). This demonstrates that the EMBV can be interpreted as the sum of the GEBV and the square root of the segregation variance, weighted by $E\left(X_{(N_G)}\right)$.

Estimates of the segregation variance can be obtained by repeatedly simulating DH progenies and calculating the sample variance of their breeding values. Alternatively, it could be calculated analytically using the well-known equation from Lynch and Walsh (1998)

$$\sigma_i^2 = 2\sum_{l}^{M} \alpha_l^2 p_l(1-p_l) + 2\sum_{l}^{M}\sum_{l'\neq l}^{M} \alpha_l^2 \alpha_{l'}^2 D_{ll'}, \tag{2}$$

where $D_{ll'}$ is the linkage disequilibrium (LD) between loci l and l' in the target population. As the goal is to calculate σ_i^2 for DH progeny, we computed $D_{ll'}$ as $D_{ll'} = (1-2r_{ll'})D'_{ll'}$. Here, $D'_{ll'}$ is the LD in the parental gametes of the candidate itself, which is 0 if the candidate is homozygous at either locus l or l', 0.25 if linkage phases are identical and -0.25 if linkage phases are reversed. The recombination fraction $r_{ll'}$ was computed from the genetic distance $d_{ll'}$ between both loci using Haldane's mapping function, which yields $D_{ll'} = e^{-2d_{ll'}} D'_{ll'}$. A similar derivation has been given by Lehermeier et al. (2017).

In Figure S2-2, we compared estimated against calculated values of σ_i for a total of $1,000$ individuals from the founder population ($N_{chr} = 20$). The correlation was high ($r \approx 0.98$) and the regression of estimated on calculated values was unbiased ($b = 1$). This was also true for other chromosome numbers ($N_{chr} = 5, 40$; data not shown). We conclude that an analytical solution following equation 2 could lead, in conjunction with equation 1, to a rapid alternative to the computationally heavy simulation-based computation of EMBV.

To illustrate how the selection of candidates depends on the value of N_G, we plotted the GEBVs of the $1,000$ individuals their value of σ_i (Figure S2-3). Lines were added that separate the top 20 candidates with highest EMBV from other individuals. These results show how with increasing N_G, candidates having a high GEBV are traded with candidates having a lower GEBV, but larger σ_i.

34 Figures

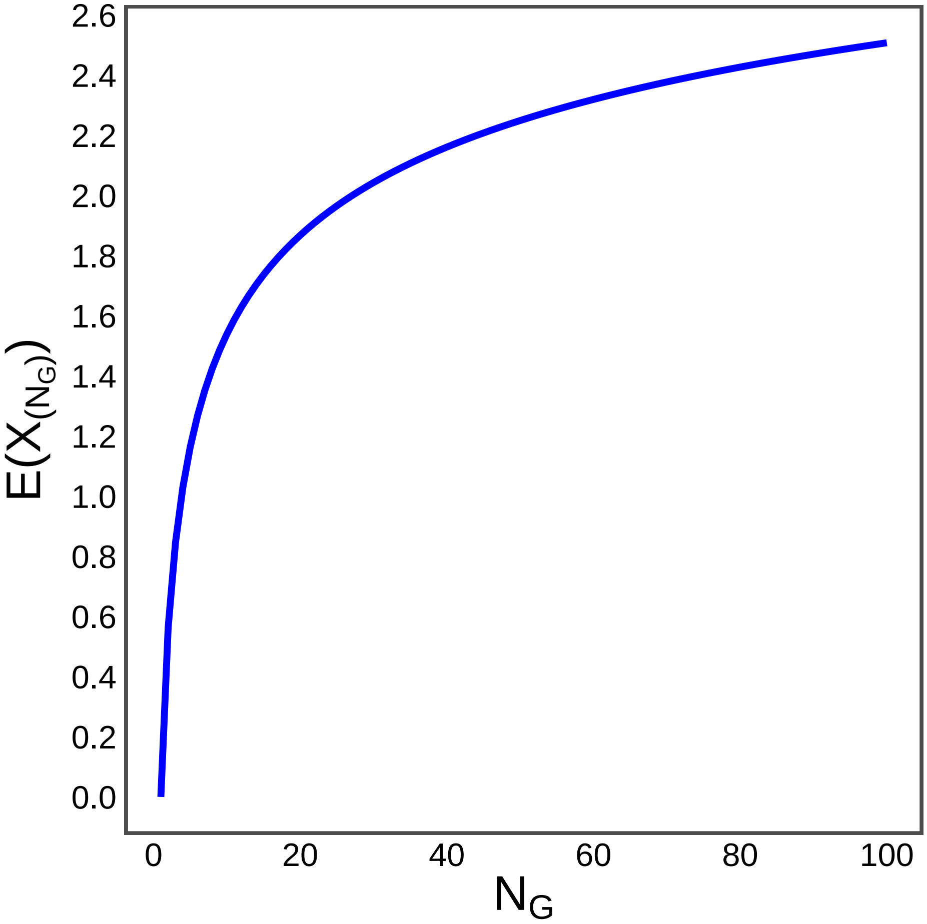

Figure S2-1: Expectation of the maximum order statistic $E\left(X_{(N_G)}\right)$ of a standard normal random variable X, depending on the sample size (number of gametes, N_G).

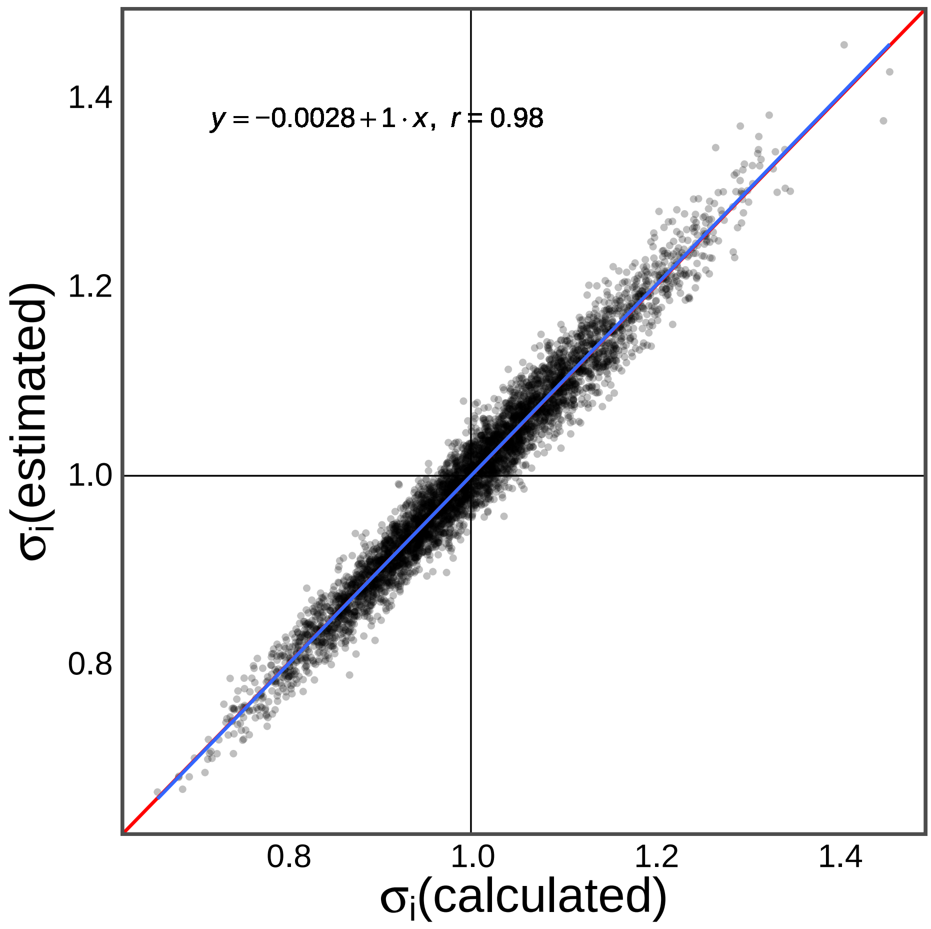

Figure S2-2: Scatter plot of estimated versus calculated square root of segregation variance (σ_i) for $1,000$ individuals from the founder population with $N_{chr} = 20$.

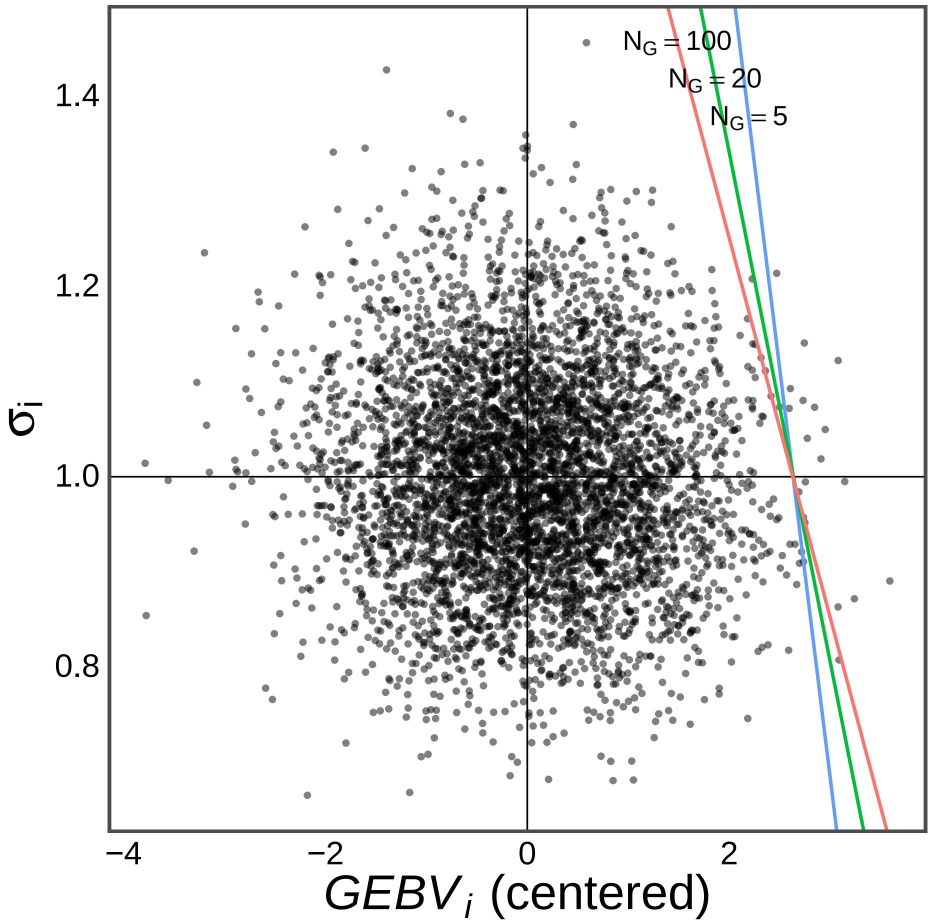

Figure S2-3: Scatter plot of genomic estimated breeding values (GEBVs) and the square root of the estimated segregation variance (σ_i) for $1,000$ individuals from the founder population with $N_{chr} = 20$. Colored lines separate selected from unselected candidates for different values of N_G, given that in each case the top 20 candidates with the highest EMBV were selected.

References

Lehermeier, C., S. Teyssèdre, and C.-C. Schön (2017). “Genetic Gain Increases by Applying the Usefulness Criterion with Improved Variance Prediction in Selection of Crosses”. In: *Genetics*.
Lynch, M. and B. Walsh (1998). *Genetics and Analysis of Quantitative Traits*. 1st edition. Sunderland: Sinauer Associates, page 980.
