# Supplementary material for: Selection on Expected Maximum Haploid Breeding Values Can Increase Genetic Gain in Recurrent Genomic Selection
Source: G3 (Bethesda). 2018 Feb 6;8(4):1173–81. doi: 10.1534/g3.118.200091 (PMC5873908; doi:10.1534/g3.118.200091)
Supplement: Supplementary file 3 [file 1173FileS3.pdf]

Selection on expected maximum haploid breeding values can increase genetic gain in recurrent genomic selection

Dominik Müller[*], Pascal Schopp[*] and Albrecht E. Melchinger[*]

[*]Institute of Plant Breeding, Seed Sciences and Population Genetics, University of Hohenheim, 70599 Stuttgart, Germany

January 28th, 2018

Correspondence: melchinger@uni-hohenheim.de

Supplemental Figures

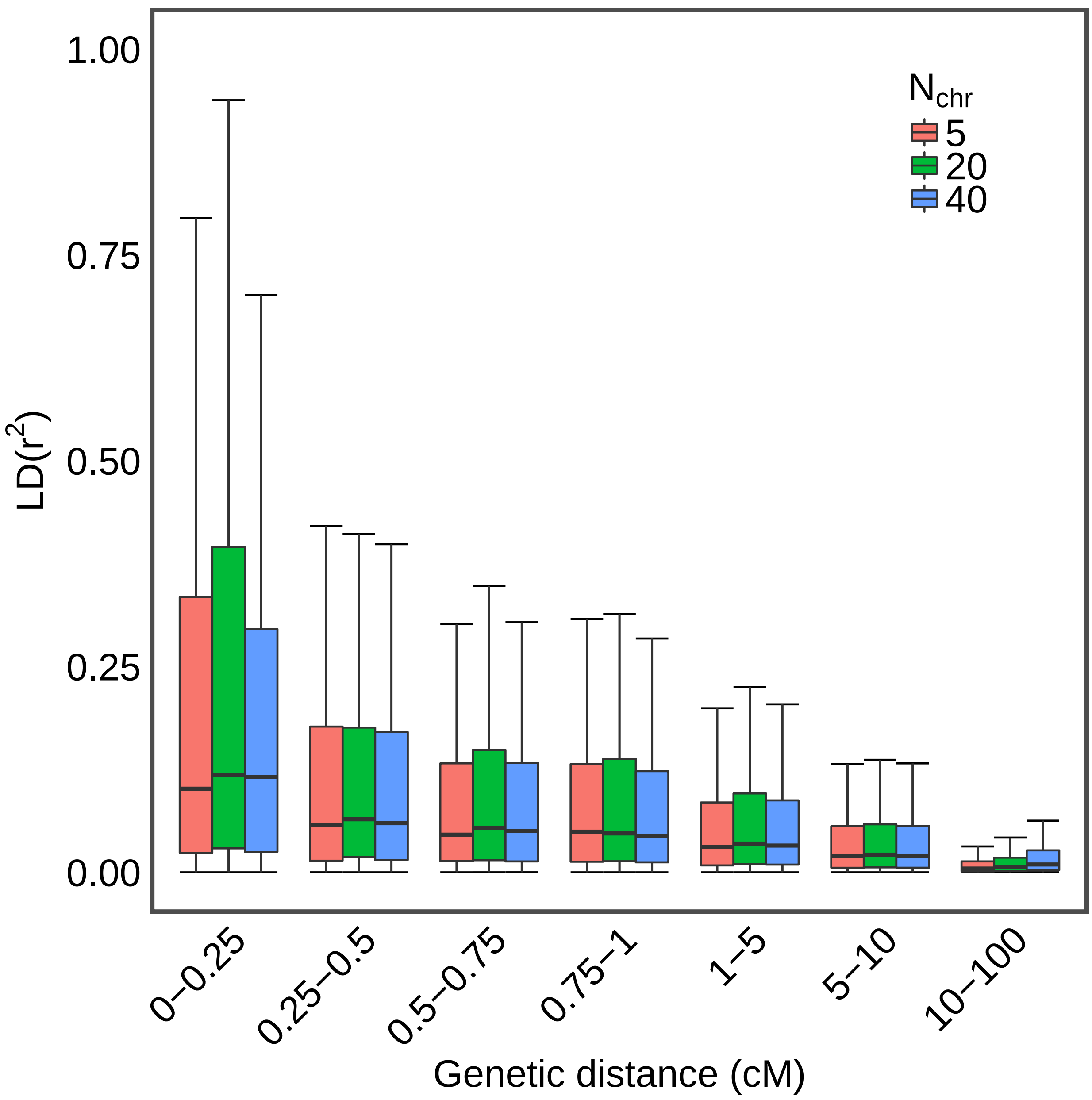

Figure S1: Distribution of pairwise linkage disequilibrium (r^2) between loci over different genetic map distances and for different base populations. N_{chr}, number of chromosomes.

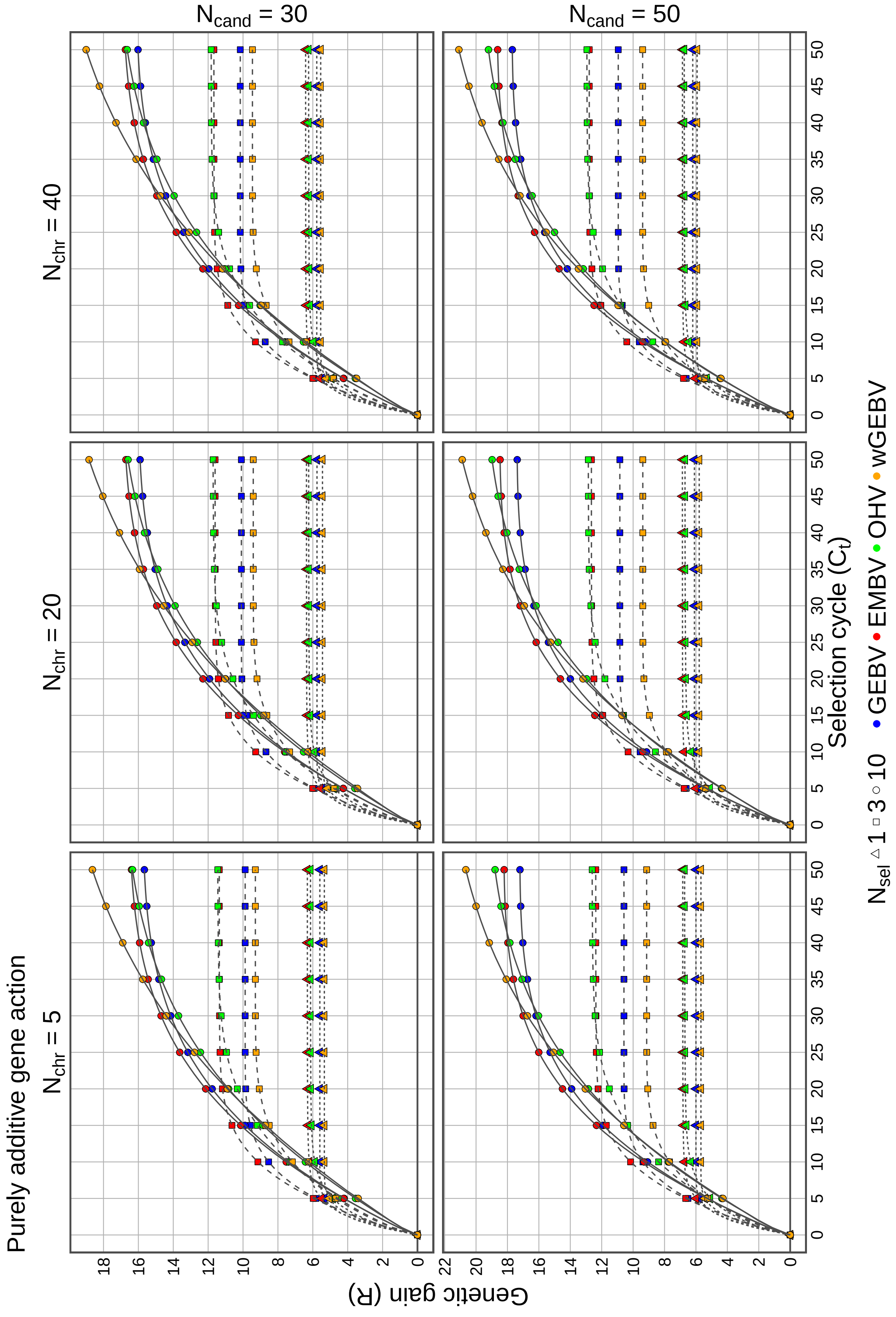

Figure S2: Genetic gain (R) for selection criteria genomic-estimated breeding value (GEBV), expected maximum haploid breeding value (EMBV), optimal haploid value (OHV), and weighted GEBV (wGEBV) under recurrent selection and purely additive gene action. N_{chr}, number of chromosomes; N_{cand}, number of selection candidates; N_{sel}, number of selected individuals.

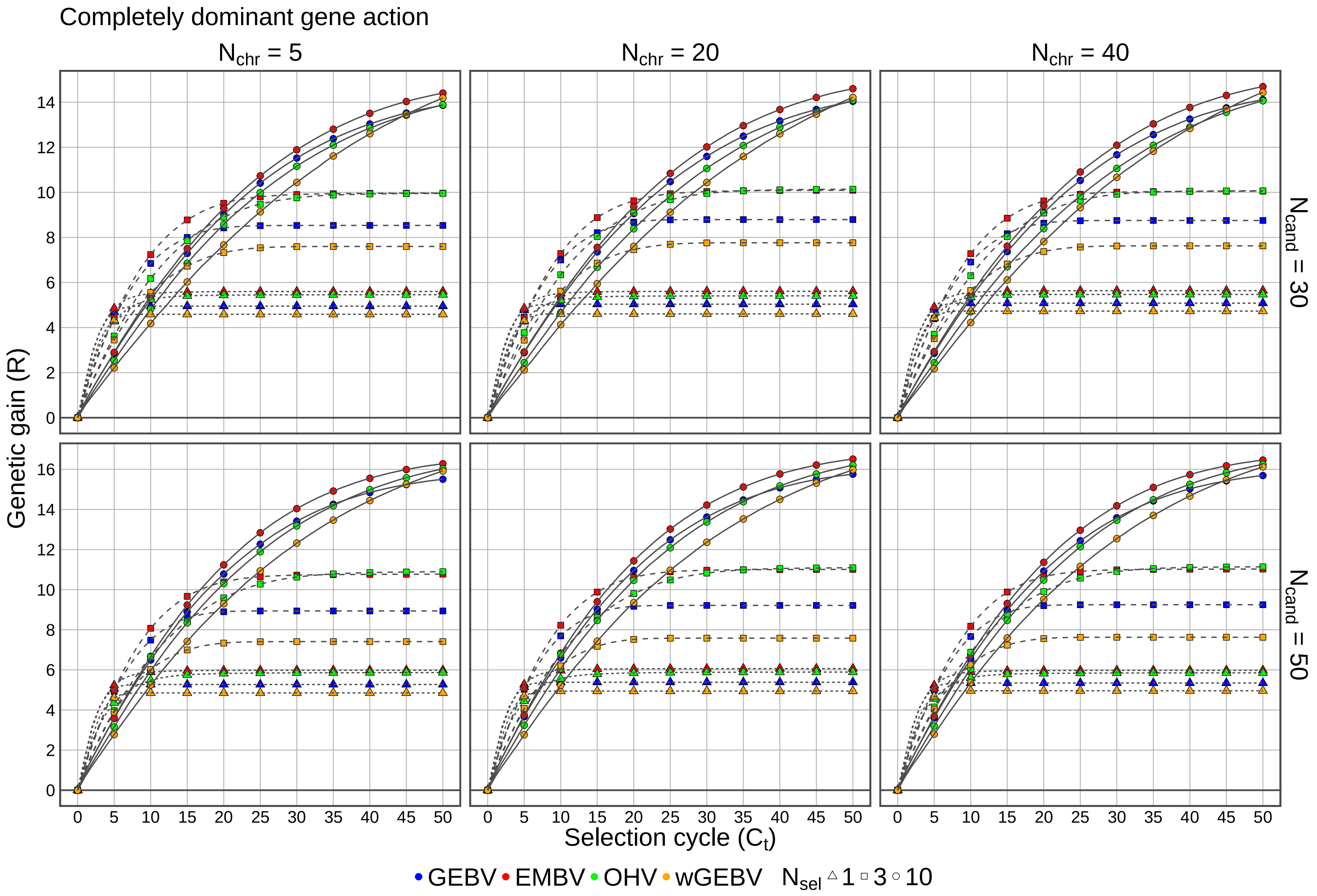

Figure S3: Genetic gain (R) for selection criteria genomic-estimated breeding value (GEBV), expected maximum haploid breeding value (EMBV), optimal haploid value (OHV), and weighted GEBV (wGEBV) under recurrent selection and completely dominant gene action. N_{chr}, number of chromosomes; N_{cand}, number of selection candidates; N_{sel}, number of selected individuals.

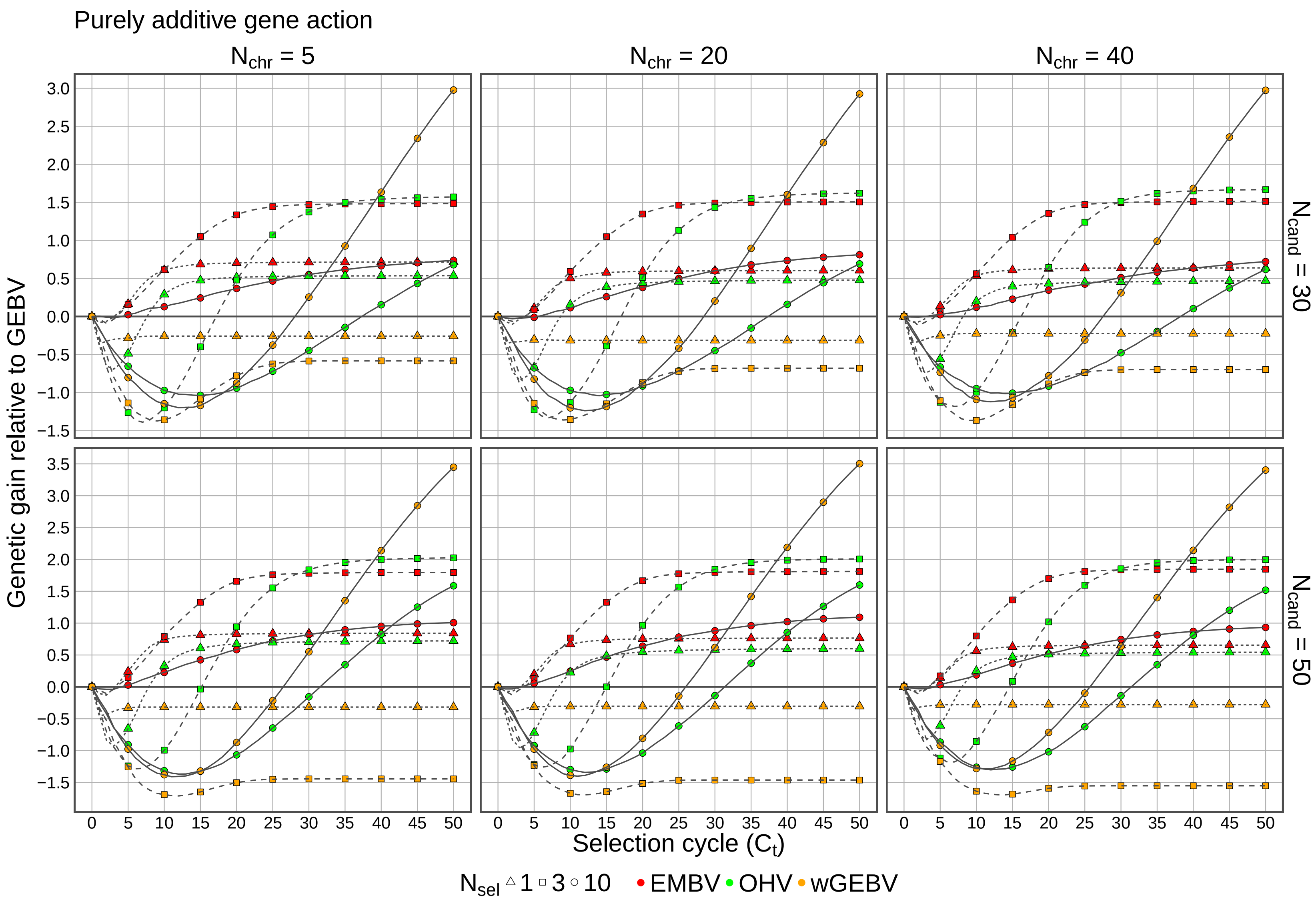

Figure S4: Difference in genetic gain (R) between selection criteria expected maximum haploid breeding value (EMBV), optimal haploid value (OHV) and weighted genomic-estimated breeding values (wGEBV) relative to GEBV under recurrent selection and purely additive gene action. N_{chr}, number of chromosomes; N_{cand}, number of selection candidates; N_{sel}, number of selected individuals.

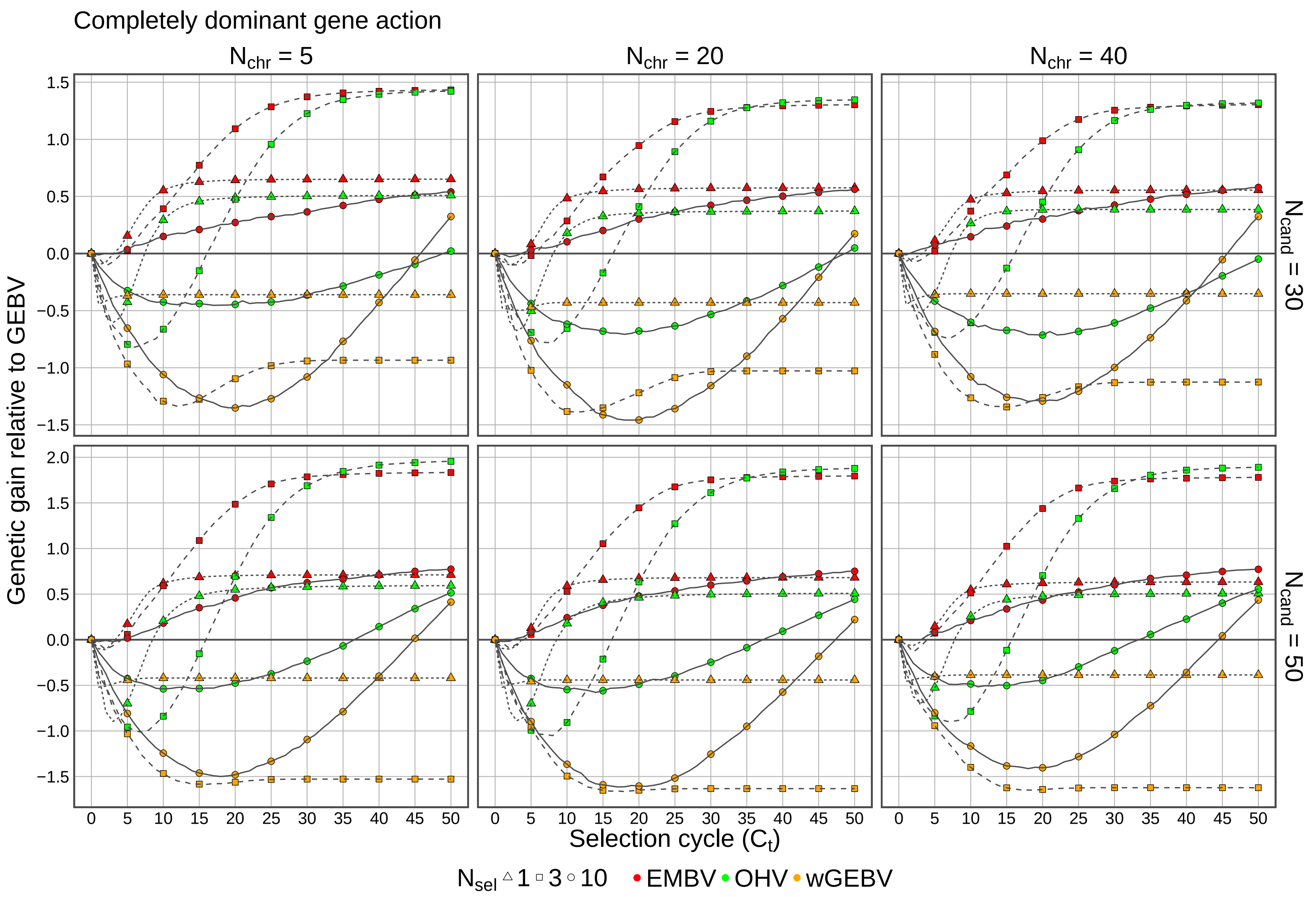

Figure S5: Difference in genetic gain (R) between selection criteria expected maximum haploid breeding value (EMBV), optimal haploid value (OHV) and weighted genomic-estimated breeding values (wGEBV) relative to GEBV under recurrent selection and completely dominant gene action. N_{chr}, number of chromosomes; N_{cand}, number of selection candidates; N_{sel}, number of selected individuals.

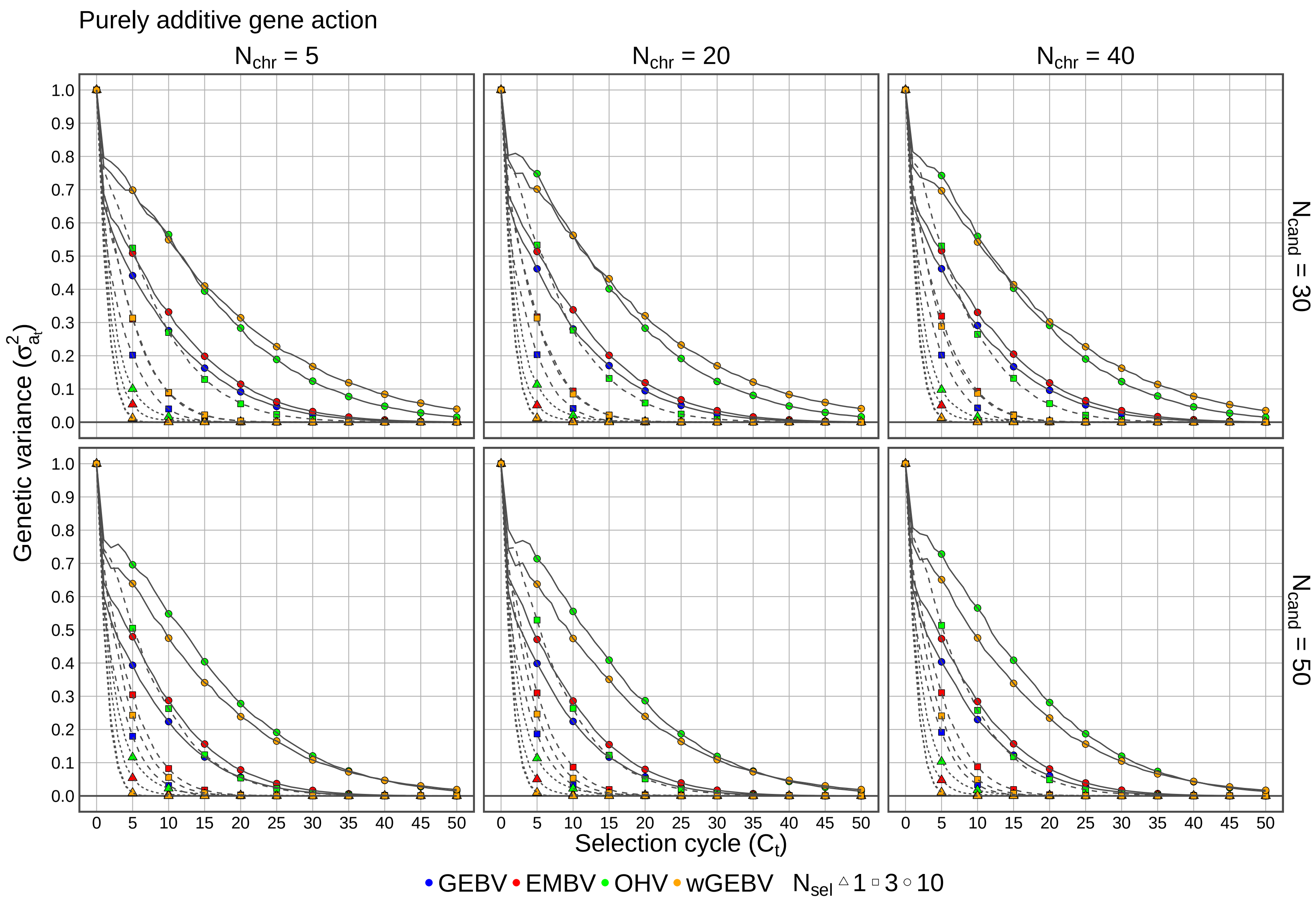

Figure S6: Additive-genetic variance ($\sigma^2_{a_t}$) for selection criteria genomic-estimated breeding value (GEBV), expected maximum haploid breeding value (EMBV), optimal haploid value (OHV) and weighted GEBV (wGEBV) under recurrent selection and purely additive gene action. N_{chr}, number of chromosomes; N_{cand}, number of selection candidates; N_{sel}, number of selected individuals.

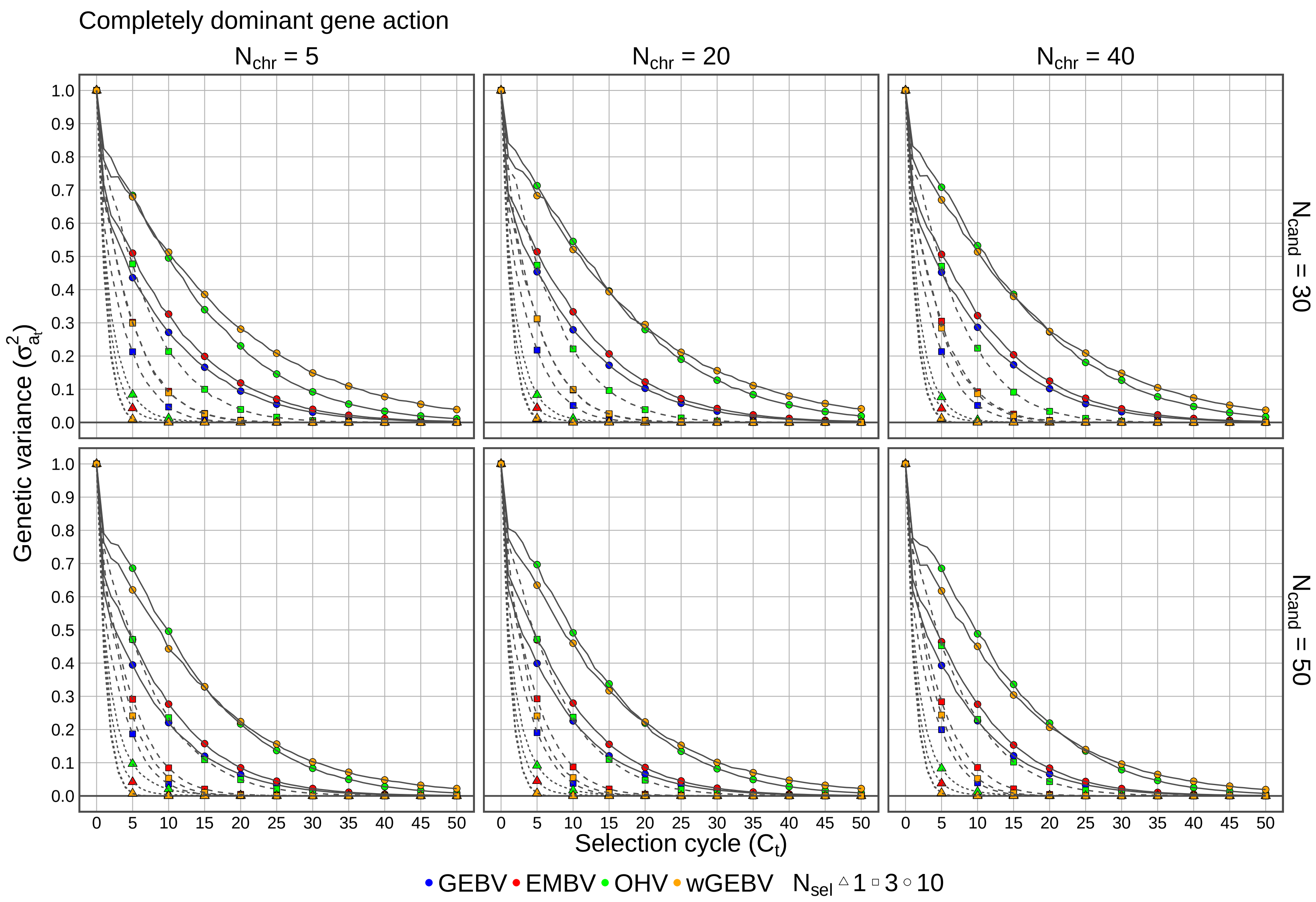

Figure S7: Additive-genetic variance ($\sigma^2_{a_t}$) for selection criteria genomic-estimated breeding value (GEBV), expected maximum haploid breeding value (EMBV), optimal haploid value (OHV) and weighted GEBV (wGEBV) under recurrent selection and completely dominant gene action. N_{chr}, number of chromosomes; N_{cand}, number of selection candidates; N_{sel}, number of selected individuals.

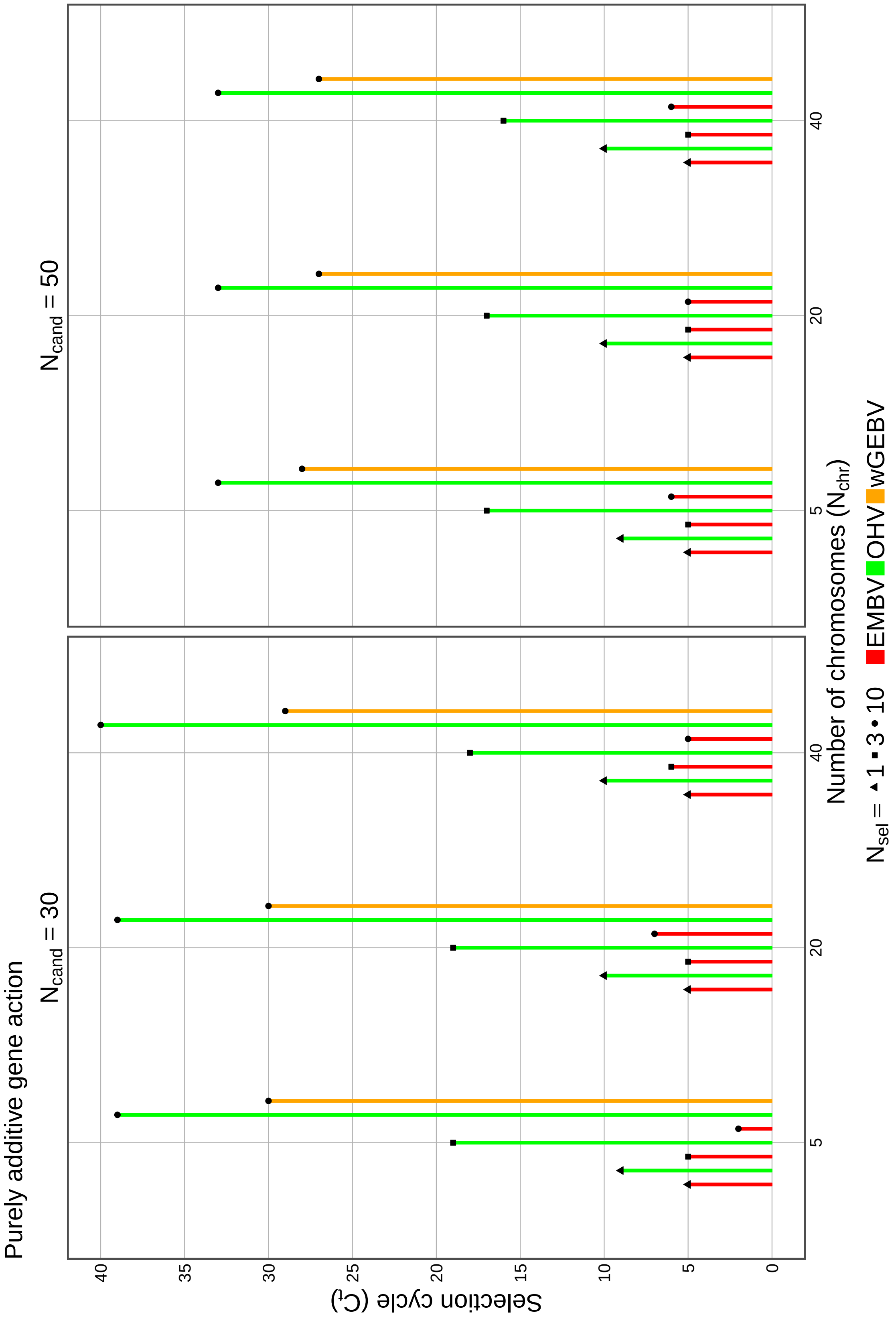

Figure S8: Selection cycle where genetic gain (R) for selection criteria expected maximum haploid breeding value (EMBV), optimal haploid value (OHV) and weighted genomic-estimated breeding value (wGEBV) surpassed selection criterion GEBV under purely additive gene action. N_{chr}, number of chromosomes; N_{cand}, number of selection candidates; N_{sel}, number of selected individuals.

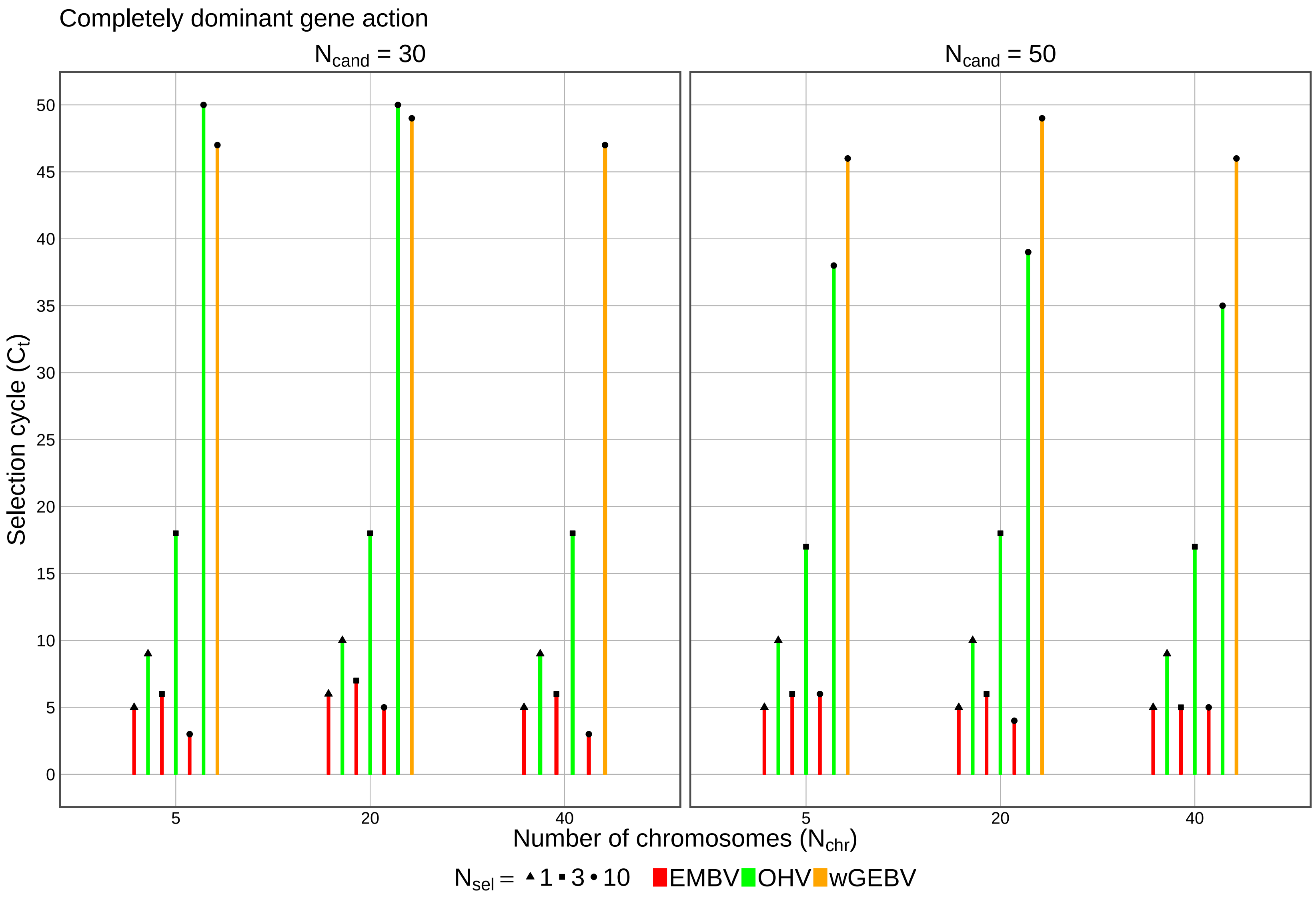

Figure S9: Selection cycle where genetic gain (R) for selection criteria expected maximum haploid breeding value (EMBV), optimal haploid value (OHV) and weighted genomic-estimated breeding value (wGEBV) surpassed selection criterion GEBV under completely dominant gene action. N_{chr}, number of chromosomes; N_{cand}, number of selection candidates; N_{sel}, number of selected individuals.

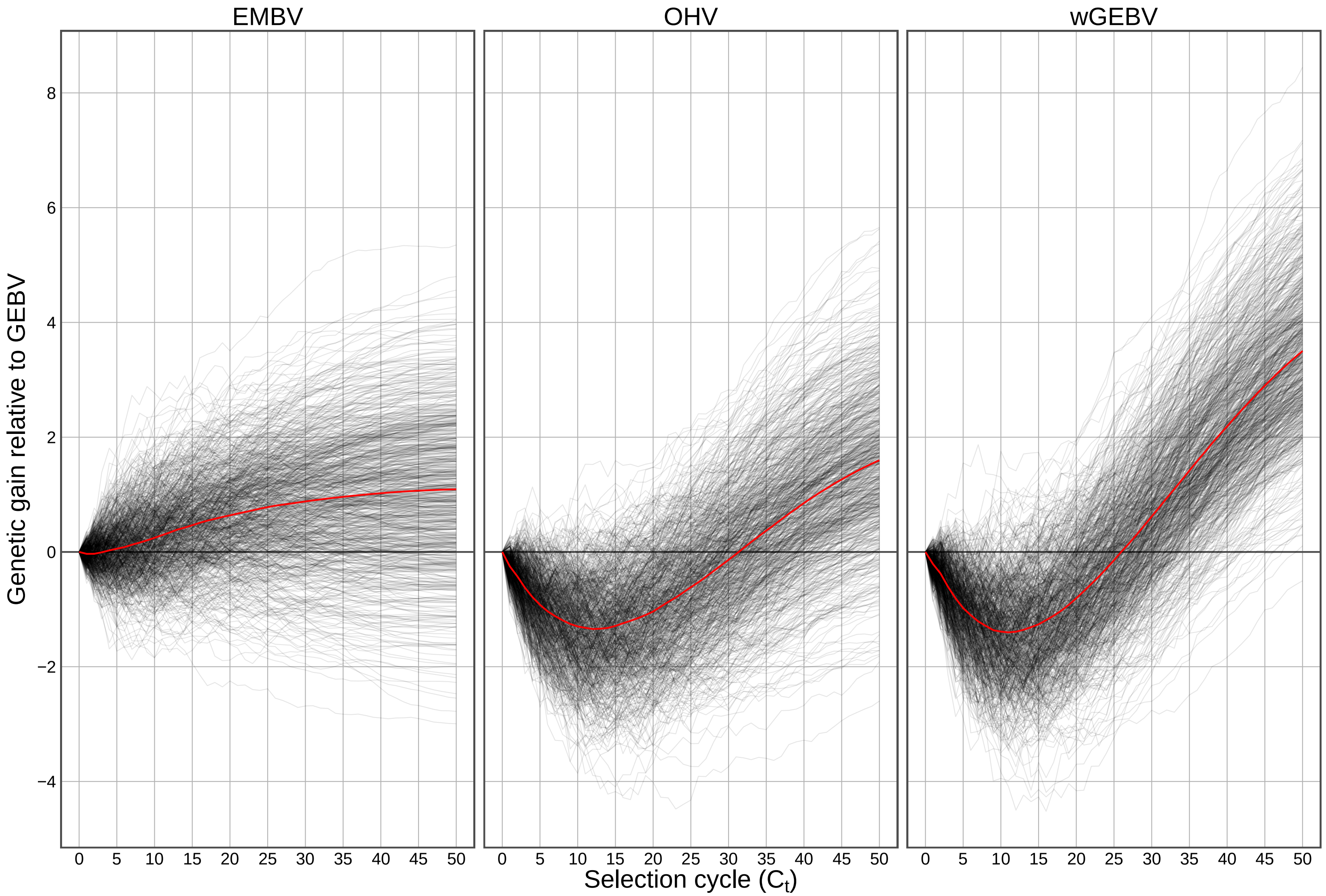

Figure S10: Trajectories of genetic gain (R) for selection criteria expected maximum haploid breeding value (EMBV), optimal haploid value (OHV) and weighed genomic-estimated breeding value (wGEBV) relative to GEBV under recurrent selection and purely additive gene action. Trajectories are shown for $N_{chr} = 20$, $N_{cand} = 50$ and $N_{sel} = 10$. N_{chr}, number of chromosomes; N_{cand}, number of selection candidates; N_{sel}, number of selected individuals.
